# Mesophilic and thermophilic viruses are associated with nutrient cycling during hyperthermophilic composting

**DOI:** 10.1038/s41396-023-01404-1

**Published:** 2023-04-08

**Authors:** Hanpeng Liao, Chen Liu, Chaofan Ai, Tian Gao, Qiu-E Yang, Zhen Yu, Shaoming Gao, Shungui Zhou, Ville-Petri Friman

**Affiliations:** 1 Fujian Provincial Key Laboratory of Soil Environmental Health and Regulation, College of Resources and Environment, Fujian Agriculture and Forestry University, Fuzhou, 350002 China; 2 Guangdong Laboratory for Lingnan Modern Agriculture, Guangzhou, 510642 China; 3 Institute of Eco-Environmental and Soil Sciences, Guangdong Academy of Sciences, Guangzhou, 510650 China; 4 School of Life Sciences, Sun Yat-sen University, Guangzhou, 510275 China; 5 Department of Biology, University of York, Wentworth Way, YO10 5DD York, UK; 6 Department of Microbiology, University of Helsinki, Helsinki, 00014 Finland

**Keywords:** Metagenomics, Microbial ecology

## Abstract

While decomposition of organic matter by bacteria plays a major role in nutrient cycling in terrestrial ecosystems, the significance of viruses remains poorly understood. Here we combined metagenomics and metatranscriptomics with temporal sampling to study the significance of mesophilic and thermophilic bacteria and their viruses on nutrient cycling during industrial-scale hyperthermophilic composting (HTC). Our results show that virus-bacteria density dynamics and activity are tightly coupled, where viruses specific to mesophilic and thermophilic bacteria track their host densities, triggering microbial community succession via top-down control during HTC. Moreover, viruses specific to mesophilic bacteria encoded and expressed several auxiliary metabolic genes (AMGs) linked to carbon cycling, impacting nutrient turnover alongside bacteria. Nutrient turnover correlated positively with virus–host ratio, indicative of a positive relationship between ecosystem functioning, viral abundances, and viral activity. These effects were predominantly driven by DNA viruses as most detected RNA viruses were associated with eukaryotes and not associated with nutrient cycling during the thermophilic phase of composting. Our findings suggest that DNA viruses could drive nutrient cycling during HTC by recycling bacterial biomass through cell lysis and by expressing key AMGs. Viruses could hence potentially be used as indicators of microbial ecosystem functioning to optimize productivity of biotechnological and agricultural systems.

## Introduction

The decomposition of organic matter is a key ecosystem process, impacting nutrient cycling and productivity across terrestrial ecosystems [1]. While it is known that both bacterial and fungal communities play crucial roles in recycling nutrients via “the microbial loop” [1], the role of bacterial viruses, i.e., bacteriophages (phages), is still poorly understood [2]. As the most abundant biological entity on Earth, viruses play a critical role in driving microbial mortality through cell lysis [3, 4], significantly impacting element cycling via release of nutrients and affecting microbial community composition, diversity and microbial necromass [5–10]. While the importance of viruses for nutrient cycling in the oceans is well established [11], we are only beginning to understand how viruses modulate the turnover of nutrients and mineralization of organic matter in soils [9, 10, 12]. Virus-encoded auxiliary metabolic genes (AMGs) associated with glycoside hydrolases and methane metabolism have been demonstrated to contribute to carbon cycling in soil ecosystems [4, 6, 13]. For example, 14 AMGs including 9 glycoside hydrolase families, such as endomannanase with confirmed functional activity, indicate that viruses have the potential capacity to participate in complex carbon degradation [4]. In addition to driving nutrient cycling via lysis of bacterial cells in soils, viruses have recently been shown to improve the survival of their host bacteria under environmental stress by encoding auxiliary metabolic genes (AMGs) that enhance the metabolic capacity of bacterial hosts [14]. Despite these recent advances, we still have a limited understanding on how viruses and bacteria together drive nutrient cycling and decomposition of organic matter across terrestrial ecosystems [15].

Here we used a hyperthermophilic composting (HTC) as a model system to study the role of mesophilic and thermophilic bacteria and their viruses in the decomposition of organic matter. HTC is a waste treatment technology used in degradation of the organic fraction of municipal or agricultural solid waste, attaining extremely high temperatures (up to 90 °C) without exogenous heating due to thermophilic bacterial community activity [16–18]. HTC contains three main temperature phases: hyperthermophilic (>80 °C), thermophilic (>50 °C) and maturation phase (ambient temperature). During the process, carbon and nitrogen-enriched polymeric substances (lignocellulose, proteins, polysaccharides and lipids) are degraded during thermophilic phases of composting, while slowly degrading humic-enriched compounds are degraded during the maturation phase. The composition of microbial communities that drive the degradation of organic matter change dynamically following the composting temperature during HTC [19], and thermophilic, heat-resistant taxa (*Firmicutes*; *Bacillus* and *Deinococcota*; *Thermus*) are important for the decomposition of organic matter during the thermophilic phase [16]. While the effects of temperature, raw materials and physicochemical composting properties have been extensively studied in relation to microbial community assembly and degradation of organic matter [20, 21], very little is known about the role of viruses during HTC.

Viruses could mediate effects on bacteria during HTC in two main ways. First, they could impose a strong top-down control on bacterial abundances via lysis as has been shown in aquatic systems [22]. Such viral predation could shape the nutrient cycling from bacterial biomass [8, 9], drive the ecological succession of mesophilic and thermophilic taxa during HTC and potentially promote bacterial diversity over time by reducing dominance effects via “Kill-the-Winner” model [23, 24]. Second, viruses could drive nutrient cycling via provision of functional genes associated with the decomposition. For example, it has been shown that viral AMGs can potentially help host bacteria to acquire nutrients and degrade toxic compounds, having positive effect on their survival [14]. Given that viruses require host cell machinery for the transcription of their genes, viral activity based on transcriptomics can be used to indicate successful infection of hosts [25]. This approach has recently been used to demonstrate that sub-seafloor sediments show activity by both lytic and lysogenic viruses [26], that the expression of AMGs are possibly involved in modulating host methane and sulfur metabolism upon infection in ocean ecosystem [22, 27] and that giant viruses are active in coastal marine system [28]. Here, we chose microbe-rich HTC as the model system to assess both bacterial and viral community composition, diversity, and activity in relation to carbon and nitrogen turnover [16]. Moreover, as HTC is typically characterized by time-dependent microbial community succession, we specifically compared the role of mesophilic and thermophilic bacteria and their DNA viruses in the dynamics of nutrient cycling.

In this study, we used an industrial-scale hyperthermophilic composting experiment to create a replicated, temporal sampling of bacteria-viral community assembly, their abundances, functional gene contents, and activity based on metagenomics and metatranscriptomics. We found that virus-bacteria density dynamics and activity are coupled, where viruses specific to mesophilic and thermophilic bacteria track their host densities, indicative of strong viral top-down control during HTC. These effects were predominantly driven by DNA viruses as the majority of detected RNA viruses (82%) were associated with eukaryotes and uncoupled from nutrient cycling during the thermophilic phase of composting. Moreover, DNA viruses specific to mesophilic bacteria encoded and expressed several AMGs linked to carbon cycling, impacting the nutrient turnover alongside with bacteria. As a result, viral abundance and activity were positively correlated with nutrient cycling, highlighting the importance of viruses for the degradation of organic matter during HTC.

## Materials and methods

### Hyperthermophilic composting and sampling

The composting experiment was carried out in a full-scale hyperthermophilic composting plant located in Shunyi district, Beijing, China (40°03′10.48″N, 116°56′2.12″E). Sewage sludge and rice husk were used as the main composting raw materials as described in a previous study [16]. Hyperthermophilic composting normally takes 45 days from start to completion and includes four temperature phases: initial phase (day 1: 32 °C), hyperthermophilic phase (from day 2 to 9: >90 °C), thermophilic phase (from day 10 to 26: >55 °C) and maturation phase (from day 27 to 45: <45 °C). To cover changes during the whole composting process, eight samples from five compost piles were collected at different phases of hyperthermophilic composting on days 0 (D0), 4 (D4), 7 (D7), 9 (D9), 15 (D15), 21 (D21), 27 (D27), 33 (D33) and 45 (D45). To obtain well-distributed and homogenized samples, each pile was diagonally divided into five domains, and each domain was sampled from the same location at a depth of 40–50 cm at different sampling time points. Within each pile, five subsamples (5000 g each) per domain were collected, and then mixed into a single composite sample, which was further divided into two aliquots. One replicate aliquot was stored in liquid nitrogen for biological analyses and the other was kept at 4 °C for physicochemical analyses. An automatic temperature controller was used to determine temperature changes during the composting.

### Determining changes in physicochemical properties during composting

Changes in the compost’s physicochemical properties were measured using previously described methods [16] unless otherwise specified. The cycling of carbon was evaluated based on total carbon content (TC), water-soluble carbon (WSC), total organic carbon content (TOC), and inorganic carbon content (IC). The cycling of nitrogen was quantified based on total nitrogen content (TN), water-soluble nitrogen (WSN), ammonium (NH_4_^+^), and nitrate (NO_3_^−^) concentrations. The TOC and IC were quantified using an automatic TOC analyzer for liquid samples (Shimadzu TOC-L CPH, Kyoto, Japan), while TN and TC were determined with Elementar instrument (Vario MAX cube, Hanau, Germany) by using dry combustion. Organic matter (OM) content was measured by dry combustion at 550 °C for 8 h. The TN and TC values were used to calculate the C/N ratio. Other measured physicochemical properties included pH, water content (WC), electrical conductivity (EC). The concentration of WSN was based on the sum of NH_4_^+^ and NO_3_^−^ contents, while WSC concentration was based on the sum of the TOC and IC.

### Analyzing changes in bacterial community composition and diversity using 16S rRNA gene amplicon sequencing

To determine changes in bacterial community composition and diversity during composting all collected samples (each time point consisting of 5 replicates) were subjected to 16S rRNA gene amplicon sequencing using a NovaSeq6000 platform (Illumina, PE250 mode, Guangdong Magigene Biotechnology Co. Ltd, Guangzhou, China). Total genomic DNA for amplicon sequencing was extracted using a DNeasy PowerSoil kit (Qiagen, Hilden, Germany) following manufacturer’s instructions. The prokaryote (bacteria and archaea) primers 515F (5′-GTGCCAGCMGCCGCGGTAA-3′) and 907R (5′-CCGTCAATTCMTTTRAGTTT-3′) targeting the V4-V5 region of the 16S rRNA gene were used. The raw 16S rRNA gene sequences were processed using *QIIME 2* (version 2019.7) [29] and quality filtered (i.e., filtered, dereplicated, denoised, merged, and assessed for chimaeras) to produce amplicon sequence variants (ASVs) using the DADA2 pipeline in QIIME2 [30]. The truncation and trimming parameters in DADA2 were set to –p-trim-left-f 0, –p-trim-left-r 0; and –p-trunc-len -f 248, –p-trunc-len-r 230. The DADA2-generated feature table was filtered to remove ASVs at a frequency less than two and the remaining ASVs were classified using the QIIME2 naive Bayes classifier trained on 99% operational taxonomic units available at the SILVA rRNA database (v 138) [31]. Microbial diversity was estimated using alpha diversity (Shannon index and observed OTU) and community composition using beta diversity (weighted UniFrac distance) based on the q2-diversity pipeline within QIIME2. To quantify the community assembly process, null model analysis was used to detect the community assembly mechanism by calculating the standard deviation between the observed ecological model and the randomly generated ecological model [32, 33].

### DNA and RNA shotgun sequencing

Three randomly selected compost replicate samples were selected for DNA and RNA shotgun sequencing at days 0, 4, 15, and 27 of composting, which represented heating, hyperthermophilic, thermophilic, and maturation phases of composting, respectively (resulting in a total of 12 metatranscriptome and 12 metagenome samples). One subset of each replicate sample was used for DNA extraction to improve the recovery of Metagenome-Assembled Genomes (MAGs), while the other was used for total RNA extraction to examine gene expression at the community level using metatranscriptomics. Prior to RNA extraction, samples were immediately stored in RNAlater (ThermoFisher Scientific) and liquid nitrogen. Total genomic DNA and RNA were extracted from 0.5 g compost samples using the DNeasy PowerSoil kit (Qiagen, Hilden, Germany) and the RNeasy PowerSoil total RNA kit (Qiagen), respectively, following manufacturers’ protocols. The samples were not filtered before metagenomic processing and hence contained both free-living viruses and intact prophages. DNA quality was assessed with a 1% agarose gel and DNA concentration was measured using Qubit dsDNA high-sensitivity assays (Thermo Fisher, Waltham, USA). RNA concentrations were measured using the Qubit RNA HS assay kit and RNA integrity determined using an Agilent 2100 Bioanalyzer (Agilent Technologies) before and after rRNA removal with Ribo-minus Transcriptome Isolation Kit (Thermo Fisher, Waltham, USA). The resulting enriched mRNA was prepared for sequencing using the TruSeq stranded mRNA library prep kit (Illumina, California, USA), following manufacturer’s protocol. The extracted DNA and cDNA from each sample were used to construct libraries (300 bp) at Guangdong Magigene Biotechnology Co. Ltd. Libraries were prepared using DNA Library Prep Kit V2 for Illumina (Illumina, California, USA) following manufacturer’s protocol, which is compatible with all DNA including dsDNA. While this kit removes most of the ssDNA viruses, a small fraction of ssDNA viruses incorporated into bacterial genomes as dsDNA or dsDNA intermediates from active replication likely remained in our libraries [34]. The sequencing was conducted by NovaSeq6000 platform (Illumina, PE150 mode, Guangdong Magigene Biotechnology Co. Ltd). The detailed information about the DNA and RNA sequencing datasets is summarized in Table S7.

### Metagenomic assembly and binning

All DNA sequences were trimmed to remove Illumina adapters and to retain high-quality reads using Trimmomatic (v 0.39, score >30 and length >36 bases) [35]. All high-quality sequences were co-assembled using SPAdes v3.13.1 with the parameters “-k 33, 55, 77, 99, 111,127 --meta” [36]. We also assembled reads generated at each thermal phase of composting separately (composting phase-specific assemblies) using SPAdes with the same parameters. All assembled scaffolds longer than 2.0 kb were binned using metawrap [37] based on MetaBAT2 [38], MaxBin2 [39], and Concoct [40] with default parameters. Bins were further manually curated to obtain high-quality genomes using Bin_refinement module in Metawrap [37]. The completeness and contamination of genome bins were assessed using CheckM v1.0.13 [41], and metagenome-assembled genomes (MAGs) with more than 50% completeness and less than 10% contamination level were retained for further analyses. Bins from different samples were dereplicated to produce medium to high quality genomes using dRep v.2.3.2 [42] and assigned to taxonomic classifications based on the Genome Taxonomy Database (GTDB; release 03-RS86) using the GTDB-Tk toolkit (v.0.3.2) with the classify workflow [43]. To construct bacterial MAGs, genes were called using Prodigal with parameters “-p meta” [44] and annotated against the KEGG and Pfam databases using the Diamond tool [45]. The predicted proteins were screened for candidate CAZymes using hmmscan module from HMMER v3.2.1 and dbCAN database (cutoffs: coverage fraction: 0.40; e-value:1e-18) [46]. Genes encoding proteases and peptidases were identified using Diamond against the MEROPS database release 12.0 (cutoffs: e-value 1e-20 -accel 0.8). Ribosomal RNAs were predicted using RNAmmer v1.2 [47]. The optimal growth temperature (OGT) of MAGs was predicted by the machine learning method using the Tome v1.1 [48]. Thermophilic MAGs were defined as ones with OGT ≥ 50 °C, while MAGs were assigned as mesophilic when their OGT < 50 °C. To build phylogenetic MAG trees, the “classify” workflow in GTDB-Tk (v.0.3.2; default settings) was used to identify 120 bacterial marker genes, which were used for tree construction based on multiple sequence alignment. The resulting FASTA files containing multiple sequence alignments of the submitted genomes were used for maximum likelihood phylogenetic tree inference using FastTree v.2.1.10 with the default parameters [49]. Newick tree output files were visualized with iTOL v.5 [50].

### Viral contig identification, taxonomic classification, and functional annotation

To identify viruses with high confidence, viral contigs larger than 5 kb were recovered from metagenome assemblies and analyzed using a combination of three tools with strict quality thresholds as previously suggested [51]. First, DeepVirFinder v1.0 [52] was ran with a loose cutoff (score 0.7 and *p* < 0.05) for maximal sensitivity to detect viral sequences. Second, VirSorter2 v2.2.1 [53] was used to identify the putative viral sequences with scores ≥0.95 using DeepVirFinder-output sequences as input files. To remove some non-viral sequences during the VirSorter2 analysis, CheckV (v0.9.0)[54] was used to quality assessment. The final viral contig dataset was manually curated and trimmed to remove potential host regions according to previous protocol [55]. Predicted contigs were considered of viral origin if they satisfied at least one of the three following criteria: (1) contigs contained at least one virus-specific hallmark gene; (2) contigs had VirSorter2 scores ≥0.95; (3) the total number of genes annotated as “unknown” (egg-NOG v5.0.0 database) accounted for ≥80% of the total number of genes on the scaffold. Finally, all potential viral contigs were further checked using VIBRANT [56] (v1.2.1, virome mode) with default settings. The identified viral contigs were clustered at 95% average nucleotide identity with at least 85% coverage using CD-HIT v4.8.1 [57] (parameters: -c 0.95 -aS 0.85), resulting in a total of 1297 viral OTUs (from here on referred as “vOTUs”). The longest sequence from each cluster was used as a representative sequence of a given viral group in subsequent analyses. Completeness of viral genomes was estimated using the CheckV pipeline. In order to determine the overlap between our representative vOTUs and viruses included in the IMG/VR v3 dataset [58], we used rapid genome clustering to identify our dataset vOTUs that shared 95% identity and 85% coverage with IMG/VR v3 viruses based on the scripts (script aniclust.py) provided in CheckV with the “--min_ani 95 --min_qcov 0 --min_tcov 85” parameters.

Taxonomic assignment of viral contigs was performed using PhaGCN2 based on the latest ICTV classification tables [59]. Reference viruses were obtained from the RefSeq viral database (v216, released in Jan. 2023). In case of unclassified viruses, CAT [60] was used for assigning viral taxonomies using the Lowest Common Ancestor algorithm against NCBI nr database. Only a very few compost vOTUs (7.7%) clustered with taxonomically known viruses based on above two methods. The non-redundant functional proteins in viral contigs were annotated (Table S8) using VIBRANT based on Pfam, dbCAN, KEGG and eggNOG databases 5.0 with default parameters [4, 61]. The phage lifestyle was predicted using three tools including VIBRANT [56], PhaTYP [62] and manually curated BLAST [25] based on previously described methods [25, 63]. Briefly, VIBRANT [56] and manual BLAST [25] were used to infer temperate lifestyle by identifying viral contigs that contained proteins associated with lysogeny (transposase, integrase, excisionase, resolvase, and recombinase) [74]. Due to incompleteness of several viral contigs, a machine learning method called PhaTYP [62] was also used to predict the phage lifestyle. Virus was inferred as a temperate phage if it was predicted to be lysogenic by any one of these tools. Additionally, vOTUs clustered with known temperate phages during vConTACT2 clustering or representing proviral sequences were assigned as temperate [25]. All other vOTUs were assigned as lytic, even though the absence of temperate phage-associated proteins could have also been due to incompleteness of viral genomes.

### Determining the relative abundance of vOTUs and MAGs in metagenomes

The relative abundances of vOTUs and MAGs in the 12 metagenome datasets (four sampling time points with three replicates each) were quantified using the CoverM pipeline [4] (v0.61, https://github.com/wwood/CoverM). The relative abundances of vOTUs and MAGs were calculated based on the coverage of mapped reads using “contig“ and “genome” mode for vOTUs and MAGs, respectively. To calculate the relative abundance of each vOTU and MAG, quality-controlled reads from each 12 compost metagenomes were mapped to the set of 1297 dereplicated viral genomes or 228 dereplicated MAGs with CoverM pipeline using “rpkm” calculation method (reads per kilobase of exon per million reads mapped). RPKM [63] is recommended for relative abundance comparisons with metagenomic datasets, because RPKM normalizes the data based on both sequence depth (per million reads) and sequence length (in kilobases). For viruses, reads after quality control were first mapped to viral contigs using “make” command in CoverM v0.6.1, to make BAM files, after “filter” command was used to remove low-quality alignments with read identity ≤95% and aligned percent ≤75% (parameters: --percentage_id 0.95 --percentage_aln 0.75). Filtered bam files were used as input in CoverM to generate coverage profiles across samples (parameters: --trim-min 0.10 --trim-max 0.90 --min-read-percent-identity 0.95 --min-read-aligned-percent 0.75 -m mean). For MAGs, the same calculation protocol was used as with vOTUs, except for using the “genome” command instead of “contig” at the last step. The coverage of each vOTU and MAG were merged as the bacterial and viral abundance matrices, which were directly used for abundance and alpha- and beta-diversity analyses (log-transformed matrices were used for Mantel correlations).

### Virus-host linkage analysis

The 1,297 vOTUs were putatively linked to 228 bacterial host MAGs using three in silico methods [4, 64] where virus-host linkages were predicted based on: (1) shared genomic content between viral and bacterial host scaffolds (bitscore ≥ 50, e-value < 10^−3^, identity ≥ 70% and matching length ≥ 2500 bp [4, 65]); (2) sequence similarity between CRISPR spacers between bacterial and viral scaffolds; (3) matching of vOTU-derived tRNA sequences in MAGs with tRNAscan (v 2.0.9, using the general and bacterial/archeal models, respectively) and BLAST (95% coverage and 90% sequence identity, e-value <1e-05) after deleting self-hits and duplicates [65]. CRISPR spacers were recovered from bacterial MAGs metagenomic contigs with CRT (v 1.2) with default parameters [66]. Extracted spacer sequences were matched with vOTUs using BLASTn (100% nucleotide identity, mismatch ≤1, and e-value ≤ 10^−5^). As OGT values of viruses could not be determined using Tome v1.1 [48], viruses were determined as thermophilic if they were associated with at least one bin with OGT ≥ 50 °C.

### Viral auxiliary metabolic gene identification and classification

Robustly identifying potential viral auxiliary metabolic genes (AMG) from metagenomes remains a challenge for the field [22, 51]. A credible potential AMG is required to have two following features [51, 63]: 1) the potential AMGs must be located between viral genes, where both the start and end regions have viral hallmark or viral-like genes; 2) the candidate AMG need to be involved in a cellular metabolic pathway. Based above principles, we used automated annotation tools DRAM-v (v 1.2.0, default parameters) to identify candidate AMGs combined with manual curation [67]. The AMG annotation file of DRAM-v was further refined using the following parameters: AMGs score of 1–3 and AMG flag of -M and -F [67]. The functional annotation of AMGs was done using three databases: the gene orthology database by eggNOG 5.0 [68], the carbohydrate-active enzyme database (CAZy) [46] (dbCAN2 HMMdb release 10.0) and KEGG database [69]. We also performed manual curation to improve the confidence of AMG identification as described previously [51, 63, 70, 71] by removing all potential illegitimate AMGs that were assigned to gene categories of DNA-related reactions, nucleotide metabolism, viral invasion (i.e., lysozymes/endolysins), modification of viral components (i.e., glycosyl transferases, adenylyltransferases and methyltransferases that putatively involved in viral DNA, RNA and structural proteins modification). This pipeline resulted in 194 high-confidence candidate AMGs from 1297 vOTUs. To further study AMG-mediated carbon metabolism, protein sequences from the retained glycoside hydrolases (GHs) were structurally modeled using Phyre2 in expert batch submission mode (http://www.sbg.bio.ic.ac.uk/phyre2/html/page.cgi?id=index) to confirm and resolve their functional predictions [72]. Of these GHs, ones with predicted secondary structure with 100% confidence score were considered in further analyses.

### Metatranscriptomics analysis of MAGs, vOTUs, and gene expression

Metatranscriptomic reads were quality filtered via Trimmomatic (v 0.39) as described previously [4]. Moreover, SortMeRNA (v4.3.4) [73] was used to remove non-coding RNA sequences (tRNA, tmRNA, 5S, 16S, 18S, 23S, and 28S rRNA sequences) from the metatranscriptomic reads. The remaining total mRNA reads in 12 compost metatranscriptomes (four sampling time points with three replicates each) were mapped back to 228 MAG or 1297 vOTU contigs to identify active bacterial and viral taxa based on the average coverage of transcripts per genome using minimap2 [74] of the CoverM pipeline (https://github.com/wwood/CoverM). Briefly, metatranscriptomic datasets were used as input reads, using the same mapping parameters as with the metagenomic read mapping, except for choosing “tpm” calculation method (Transcripts Per Kilobase Per Million Mapped Reads, TPM). All activity was quantified at the level of vOTUs and MAGs and viruses and bacteria were deemed as active when the TPM values were larger than 0 for both duplicates of two out of three biological replicates (TPM values for each MAG and vOTU is provided in Supplementary Table 4). As viruses contained only one contig (one vOTU), they were deemed as active even if one viral gene showed TPM values larger than 0. To determine the expression of annotated genes in assembled MAGs and vOTUs, mRNA reads were mapped to a concatenated Fasta file including all genes of MAG bin or vOTU using Hisat2 with default parameters [75]. Quantification of mapped reads per identified gene was performed with the function featureCounts of the R Subread package [76]. The transcript abundance of each gene or contig was converted to transcript per million (TPM, Eq. (1)) for each sampled depth.

1 $${{{{{{{\mathrm{TPM}}}}}}}} = {{{{{{{\mathrm{A}}}}}}}} \ast {{{{{{{\mathrm{1}}}}}}}}/\sum {{{{{{{\mathrm{A}}}}}}}} \ast {{{{{{{\mathrm{10}}}}}}}}^{{{{{{{\mathrm{6}}}}}}}}$$

where *A* = reads mapped to gene/gene length (kbp).

### Metatranscriptomics analysis of RNA viruses

As recent studies have suggested that RNA viruses could also be important but overlooked players underlying the ecosystem functioning [77–80], we studied changes in their diversity and composition during composting following methods reported in previous studies [78, 79, 81]. Briefly, we used the RNA-dependent RNA polymerase (RdRp) hallmark gene as the target gene to study RNA viruses during composting (a total of 12 metatranscriptome samples, including four time points and three replicates). All assembled contigs (>1000 bp, composting phase-specific assemblies) were compared against the database containing all available viral RdRp gene sequences in NCBI/GenBank (37, 441 genes, downloaded on February 2023) and previous published studies [77, 78] using Diamond BLASTx (coverage ≥ 70%, E-value≤1e-10 and score ≥ 70). Sequences that had hits in the RdRp database with the RdRp core domain were considered as the potential RNA viruses [80]. This analysis identified 109 contigs with RdRp gene. These potential RNA virus contigs were clustered with CD-HIT using 95% average nucleotide identity across 85% alignment fraction, resulting in a total of 83 potential RNA viruses.

We also analyzed the presence of ssDNA phages in assembled metatranscriptomic contigs using the same methods as with other viruses (except for VIBRANT tool). A total of 68 putative dsDNA phage contigs (with sizes >5 kb) were obtained from the metatranscriptomic assemblies. After clustering (95% nucleotide similarity and over 85% coverage), a total of 41 dsDNA viral operational taxonomic units (dsvOTUs) were retained. By comparing the viruses’ contigs derived from transcriptomic data and metagenomic data, only 7 of 68 dsvOTUs could be assembled from the metagenomic data. This is not surprising as the DNA was removed during RNA library preparation and very few DNA sequences was retained in transcriptome.

### Statistical analyses

Data were statistically analyzed using the R platform v 3.6.1(https://www.r-project.org/) [82]. The microbial alpha and beta diversity analyses (including alpha index and PCoA), were conducted using vegan and ggplot2 packages in R. The overall mean differences across sampling time were analyzed using one-way ANOVA followed by multiple comparisons using Tukey HSD test using the *p*-value smaller than 0.05 as significance threshold. Nonparametric PERMANOVA (Adonis function) was used to determine the significance of sampling time points on the microbiome composition. In case of non-parametric Wilcoxon signed-rank and Adonis tests, statistical significance was determined based on 999 permutations. Differential gene expression between metatranscriptomes was analyzed using DESeq2 with FDR correction at *p* = 0.05.

#### MAG and vOTU diversity analyses

To evaluate the diversity of each sample, the alpha-diversity (richness, Shannon’s index) was assessed using the package “vegan” (https://cran.r-project.org/web/packages/vegan). Beta-diversity was quantified using the two first axes of Bray–Curtis dissimilarity matrix. Statistical significances between sample groups were tested using a PERMANOVA with 999 permutations.

#### Analyzing MAG and vOTU community composition using partial Mantel tests

A partial Mantel test was performed to assess the correlation between two multivariate matrices while controlling for the potential effects of nutrients turnover (carbon and nitrogen) using the R package “vegan”. Distances for vOTU and MAG abundances and nutrient turnover during composting were calculated using the Bray–Curtis dissimilarity matrices. Partial Mantel correlations were computed between all pairs of distance matrices for MAGs and vOTUs with 999 permutations for each comparison. The false discovery rate was computed using the Benjamini–Hochberg method.

#### Random Forest modeling regression analysis for predicting nutrient cycling during composting

To identify the major predictors explaining the nutrient cycling during composting, we compared the contribution of bacterial and viral community composition (based on Bray–Curtis dissimilarities) at the DNA and RNA level using default parameters of the Random Forest (RF) analysis [83]. In these RF models, the importance of each microbial predictor (bacterial and viral community composition at DNA and RNA level) was determined for the compost nutrient turnover (Euclidean distance dissimilarity based on all physicochemical composting properties). To assess the relative importance of different predictor variables, we compared the percentage increases in MSE (mean squared error), where high MSE percentage values denote for relatively more important contribution by given predictor variables [84]. Significance of the models and cross-validated *R*^2^ values were assessed with 1000 permutations of the response variable using the “A3” package in R. The analysis was conducted using the “rfPermute” package in R.

#### Partial least squares path modeling analysis for predicting nutrient cycling during composting

Partial least squares path modeling (PLS-PM) was employed to explore the direct and indirect effects of different types bacterial (Bray–Curtis dissimilarity of mesophilic (OGT < 50 °C) and thermophilic MAGs (OGT ≥ 50 °C) and mesophilic and thermophilic viral community composition (Bray–Curtis dissimilarity of vOTUs) and catabolic activity (metatranscriptomics of MAGs and vOTUs) in relation to carbon and nitrogen cycling during composting [85]. The carbon cycling index was determined as temporal change in dissimilarity matrix (based on Euclidean distance) for TC, OM, IC, WSC, and TOC, while nitrogen cycling index was based on temporal change in dissimilarity matrix (based on Euclidean distance) for TN, C/N, WSN, NH_4_^+^, and NO_3_^-^. The final PLS-PM model was chosen based on the Goodness of Fit (GoF) statistic—a measure of the model’s overall predictive power. PLS-PM was analyzed using R v3.6.1 via the package “plspm” (v 0.4.7).

## Results

### Characterization of composting properties and decomposition of organic matter during HTC

**Fig. 1:**
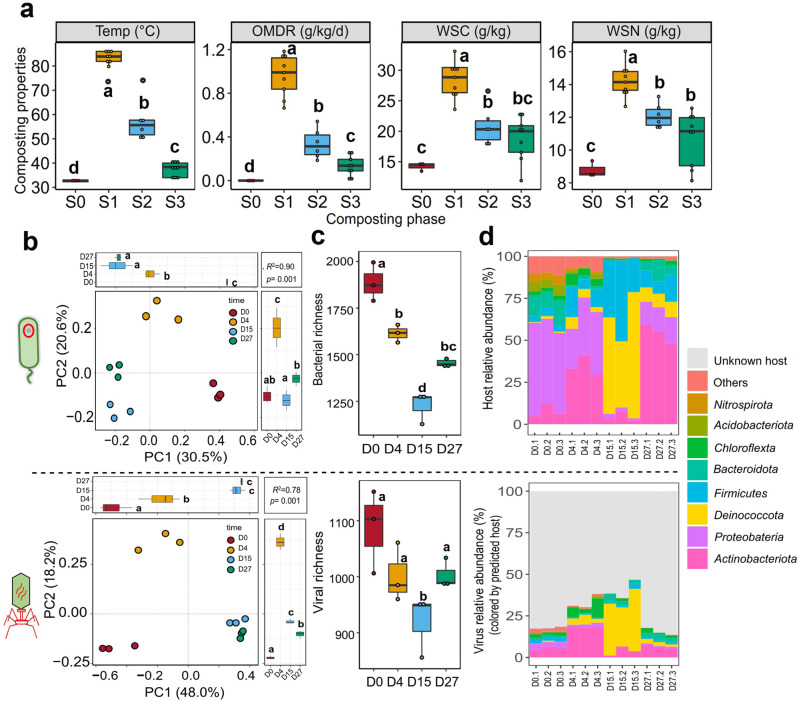
The overview of composting properties, bacterial and viral diversity, and composition during HTC. **a** Changes in composting properties during different phases of HTC. S0: initial phase (D0); S1: hyperthermophilic phase (D4 to D9); S2: thermophilic phase (D15 to D21); S3: maturation phase (D27 to D45). Temp: composting temperature; OMDR: organic matter degradation rate; WSC: water-soluble carbon; WSN: water-soluble nitrogen. **b** Changes in bacterial (upper panel) and viral (lower panel) community composition during different phases of HTC based on D0, D4, D15, and D27 sampling time points. **c** Changes in bacterial (upper panel) and viral (lower panel) species richness during HTC. **d** Changes in the relative bacterial (upper panel; phyla level) and predicted viral host taxa abundances during HTC (lower panel; based on metagenome read mapping; *n* = 12). In (**a**) and (**c**), data show mean ± SD with three biological replicates per treatment (*n* = 3); different lowercase between treatments denotes significant differences at *p* < 0.05. All datasets are based on metagenomics.

Changes in composting properties were quantified during a 45-day full-scale HTC experiment focusing on temperature, organic matter decomposition, and carbon and nitrogen content. The temperature rapidly increased to around 90 °C after 2 days of fermentation and remained >80 °C for 9 days (“hyperthermophilic phase”), after gradually declining to 55 °C (“thermophilic phase”) and ambient temperature by day 27 (“maturation phase”, Fig. 1a). The organic matter (OM) decomposition, carbon, and nitrogen turnover followed closely different phases of HTC (Fig. 1a). Compared to the initial composting raw materials, total carbon (TC, *F*_3,23_ = 33.6, *p* < 0.0001) and nitrogen (TN, *F*_3,23_ = 19.8, *p* < 0.0001) contents significantly decreased by 32% and 28% by the end of HTC, respectively (Fig. S1a). Similarly, the OM content that showed the highest degradation rate at the hyperthermophilic phase declined from 51.3% to 38.7% (*F*_3,23_ = 68.3, *p* < 0.0001), while the concentration of water-soluble carbon (WSC, *F*_3,23_ = 19.8, *p* < 0.0001) and water-soluble nitrogen (WSN, *F*_3,23_ = 26.4, *p* < 0.0001) increased during HCT, reaching peak concentrations at the hyperthermophilic phase (Fig. 1a). The degradation rate of OM correlated positively with temperature, WSC, and WSN (Fig. S1b), indicative of efficient nutrient cycling during HTC.

### Bacteria-virus community dynamics are coupled with different phases of HTC

To link changes in nutrient turnover with microbial community dynamics, we characterized bacterial and virus community diversity and composition during HTC using metagenomic and amplicon sequencing. Of all the metagenomic reads, bacterial sequences accounted between 50 and 60%, while less than 1% could be assigned to the archaea and eukaryotes (fungi, protozoa, and algae) and less than 0.1% to viruses. Based on 16S rRNA gene sequencing, more than 11 bacterial phyla (*Acidobacteriota*, *Actinobacteriota*, *Armatimonadota, Bacteroidota*, *Chloroflexota*, *Deinococcota*, *Gemmatimonadota*, *Firmicutes*, *Nitrospirota*, *Planctomycetota*, and *Proteobacteria*) were detected during the HTC, with *Firmicutes*, *Acidobacteriota*, *Bacteroidota*, *Deinococcota*, and *Proteobacteria* being the most dominant phyla by accounting for 92.5% of all taxa (Fig. S2a). While these phyla were consistently present at all stages of HTC, they showed substantial changes in their abundances (Fig. S2a). Bacterial communities were further analyzed based on the metagenomic dataset. Composting temperature had significant effects on bacterial richness (*F*_3,8_ = 44.1, *p* < 0.001) and composition (*R*^2^ = 0.89, *p* < 0.001, PERMANOVA test, Fig. 1b, c). *Proteobacteria* (51.4%) and *Bacteroidota* (12.0%) were the most dominant phyla at the initial phase of composting, while thermophilic *Thermus* and *Planifilum* genera belonging to *Firmicutes* and *Deinococcota* significantly increased in relative abundances from 5.3% at D0 to 91.4% by D15 (*F*_3,8_ = 25.8, *p* < 0.001, Fig. 1d). Maturation phase (D27) of HTC was associated with high relative abundances of *Actinobacteriota*, including *Actinomadura* and *Streptomyces* genera (*F*_3,8_ = 72.6, *p* < 0.0001). Both bacterial taxa abundances (based on phylum level) and community richness clearly followed the different phases of HTC composting (Fig. 1b, c; qualitatively similar changes observed based on 16S rRNA gene amplicon sequencing, Fig. S2). The bacterial community assembly (based on βNTI index from amplicon sequencing) correlated with cycling of carbon and nitrogen and composting temperature (all *p* < 0.0001, Fig. S3), indicative of temperature-dependent, deterministic compost community assembly.

**Fig. 2:**
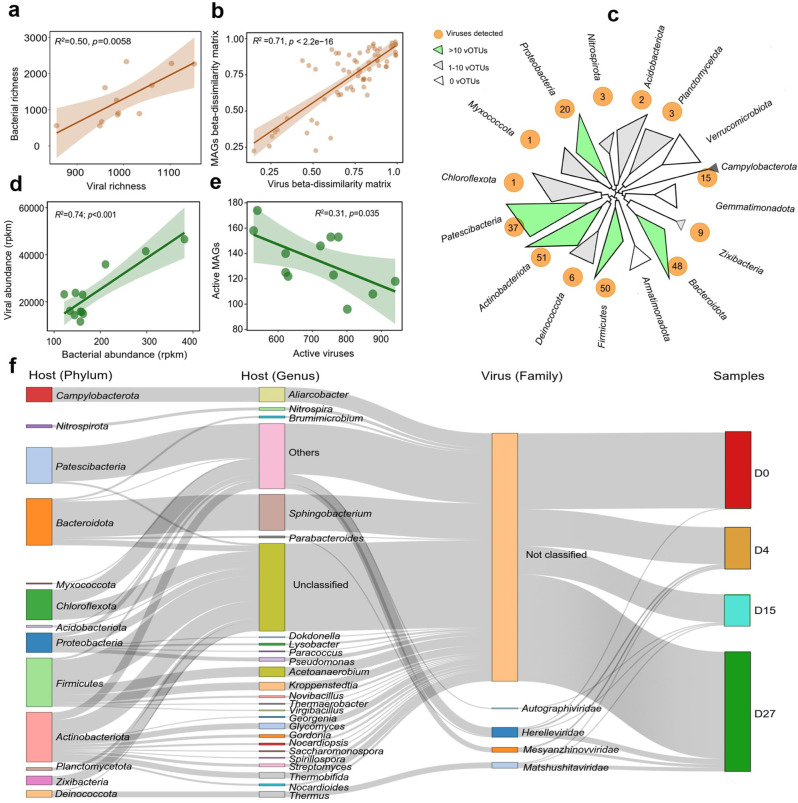
Bacteria-virus linkages and coupling of metagenome-assembled genome (MAG) and vOTU dynamics during different phases of HTC. The relationship between viral and bacterial communities based on richness (**a**) and beta-diversity indexes (**b**). **c** Phylogenetic tree of MAGs (phylum level) recovered from metagenomes based on a concatenated set of 120 conserved bacterial single-copy marker genes. The orange circles denote for MAG lineages that were predicted to be infected by viruses, with the numbers of identified vOTUs shown inside the circles. The tree scale shows nucleotide substitutions per site. **d** Positive correlation between viral and predicted host bacterial abundances across all composting samples. **e** Negative correlation between the detected number of active viruses and active host bacteria across all composting samples. (f) Predicted virus-host links between viral taxonomy (at family level) and bacterial MAGs during HTC. The left two panels represent host taxonomy colored by phylum and genus, and the right two panels show viral clusters associated with sampling days. Gray connecting lines show associations between bacterial host (at phylum and family level) and viruses associated with given sampling time points on the right. In (**a**–**e**), shaded area shows 95% confidence interval around the fitted mean line.

We first focused on analyzing changes in DNA viral communities during composting. A total of 1507 putative viral contigs (with sizes >5 kb) were obtained from the metagenomic assemblies. After clustering (95% nucleotide similarity and over 85% coverage), a total of 1297 viral operational taxonomic units (vOTUs) were retained (Table S1), which mainly belonged to double-stranded DNA viruses (97%) and were predicted to be mostly lytic (66.2%). The genome quality of vOTUs consisted of 0.7% of high-quality, 2.4% of medium-quality, and 85.6% low-quality vOTUs, while the quality of remaining 11.3% of vOTUs could not be determined. Overall, 78.6% of vOTUs were detected during non-thermophilic phases (D0 and D27), while 21.3% occurred during thermophilic phases (D4 and D15, Table S1). Only 7.7% of vOTUs could be clustered with taxonomically known viruses in RefSeq database (v216, released in February 2023), while only 35 vOTUs (2.6%) clustered with known viruses in IMG/VR (v3) database, suggesting that most of the composting viruses were novel. Primarily, they belonged to *Dividoviricota* (88%) and *Uroviricota* (2%) phyla and *Mesyanzhinovviridae* (27.2%), *Herelleviridae* (18.2%), *Salasmaviridae* (16.4%), *Autographiviridae* (5.4%), *Vilmaviridae* (5.4%) and *Matshushitaviridae* (3.6%) families (Table S1 and Fig. S4). Similar to bacteria, the richness (*F*_3.8_ = 4.7, *p* = 0.0359) and composition (*R*^2^ = 0.78, *p* < 0.001, PERMANOVA test) of viral communities followed different phases of HTC (Fig. 1d). While *Vilmaviridae* (37.8%) and *Autographiviridae* (14.5%) were dominant viruses in the composting raw material (D0), *Vilmaviridae* abundances significantly decreased to 1.5% by the maturation phase (D27; *F*_3,8_ = 9.7, *p* = 0.0047, Fig. S4). In contrast, relative abundance of *Matshushitaviridae* family under *Dividoviricota* phylum (consisting mainly of thermophile-associated *Thermus* phages) increased from 1.4% at D0 to 66.3% at D15 (*F*_3,8_ = 5.7, *p* = 0.0245, Fig. S4). As most of viruses could not be classified, viral abundances were also investigated based on their predicted host taxonomy (see Methods). The viral taxa abundances followed bacterial taxa abundances (Fig. 1d), and for example, the abundance of viruses infecting *Deinococcota* clearly increased with rising composting temperature by D15. Moreover, *Matshushitaviridae* viral abundances correlated positively with their *Firmicute* (*R*^2^ = 0.34, *p* = 0.028) and *Deinococcota* (*R*^2^ = 0.53, *p* = 0.0042, Fig. S5) host abundances. Overall, changes in viral community richness (*R*^2^ = 0.50, *p* = 0.0058) and composition (beta-dissimilarity, *R*^2^ = 0.71, *p* < 0.0001) correlated positively with changes in bacterial community richness and composition (Fig. 2a, b).

To study virus-host dynamics in more detail, we reconstructed prokaryotic metagenome-assembled genomes (MAGs) for bacteria by binning shotgun metagenomic contigs. In total, 515 medium to high-quality MAGs could be assembled (with estimated completeness of ≥50% and contamination ≤10%) across all samples, which included 513 bacteria and 2 archaea. De-replication within each time point reduced the total number of bacterial MAGs to 227 (17 phyla, Fig. 2c), including 180 mesophilic (OGT < 50 °C) and 47 thermophilic (OGT ≥ 50 °C) MAGs based on their predicted optimal growth temperatures (OGT, Table S2). The taxonomic composition and abundances of MAGs at phylum level were similar to the results based on 16 S rRNA gene sequencing data (Fig. S6), which suggests that MAGs were representative of the total bacterial diversity. To associate bacterial MAGs with viruses, three different in silico approaches were used: sequence similarity matching, CRISPR spacer linkages, and tRNA matching between MAGs and vOTUs (see Methods). We found that 21.3% of compost vOTUs were associated with putative MAG hosts based on above three methods (Table S3). Of these viruses, only 6.8% vOTUs were taxonomically assigned to *Mesyanzhinovviridae* (24.0%) and *Herelleviridae* (22.0.6%) families. Commonly predicted hosts included *Actinobacteriota* (18.4% of virus-host pairs), *Firmicutes* (18.05%), *Bacteroidota* (17.3%), *Patescibacteria* (13.3%), *Chloroflexota* (11.2%), *Proteobacteria* (7.2%) and *Deinococcota* (2.5%), which also were the most abundant bacteria during HTC (Fig. 2c–f). The viral hosts also included thermophilic bacteria with predicted OGT higher than 50 °C: *Thermus thermophilus* (T_bin.227, OGT = 69.8 °C), *Planifilum fulgidum* (T_bin.201, OGT = 58.9 °C) and *Limnochordales* (D15-1-bin.18, OGT = 70.1 °C). Viruses with predicted hosts accounted for 15–33% of the total viral community, and 45% putative hosts were associated with more than one virus, indicative of polyvalence (Table S3). Viral and bacterial abundances (linear regression, *R*^2^ = 0.74, *p* < 0.001) and community composition (Mantel statistic *r*: 0.71, *p* < 0.001) correlated positively with each other (Fig. 2d), while the number of active MAGs and vOTUs correlated negatively with each other (Fig. 2e). Together, these results suggest that bacterial and viral community dynamics were tightly coupled during HTC.

### Bacterial and viral activity are associated with carbon and nitrogen cycling during HTC

To link changes in bacteria-virus dynamics with total microbial community functioning during the HTC, we annotated all the genes we could detect using unigenes approach (2,485,700 genes) [86] and assigned them to different functional categories based on KEGG Orthology database. A total of 38.2% of all genes (950,304) could be annotated, whose abundances followed different phases of HTC. Especially, genes linked to microbial metabolism (carbohydrate metabolism, lipid metabolism, amino acid metabolism and metabolism of cofactors and vitamins) were significantly (*p* < 0.05) enriched by 17% at thermophilic phases of HTC (Fig. S7a). In contrast, the relative abundance of genes associated with nitrogen metabolism, quorum sensing, and two-component regulatory systems was significantly reduced by 19% (*p* < 0.05) during the thermophilic phase of HTC (D15, Fig. S7b).

**Fig. 3:**
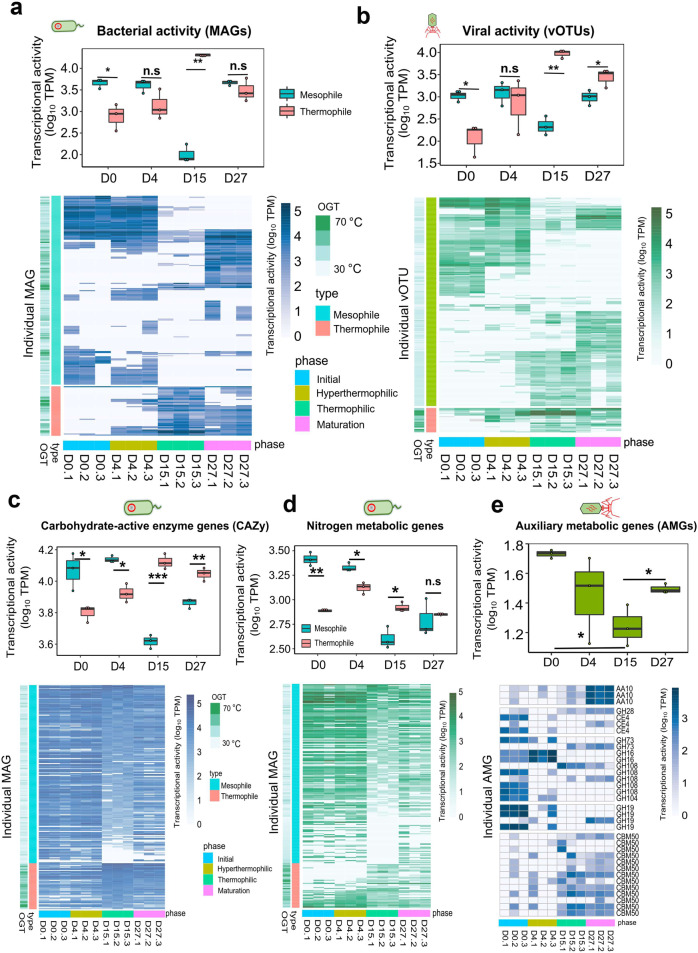
Comparison of metabolic activity of mesophilic and thermophilic bacteria (MAGs) and viruses (vOTUs) during HTC based on transcriptional activity of functional genes. **a** Box plots and heatmap representing changes in transcriptional activity of mesophilic (OGT < 50 °C) and thermophilic (OGT > 50 °C) bacteria (upper panel) and 180 mesophilic and 47 thermophilic MAGs (lower panel) during HTC. **b** Box plots and heatmap representing changes in the transcriptional activity of viruses associated with mesophilic and thermophilic bacteria (upper panel) and individual vOTUs (lower panel) during HTC. Box plots and heatmaps representing changes in the transcriptional activity of mesophilic (OGT < 50 °C) and thermophilic (OGT > 50 °C) bacteria during HTC based on mean (upper panel) and individual (lower panel) MAGs (including 180 mesophilic and 47 thermophilic MAGs) in association to carbon (CAZyme) (**c**) and nitrogen metabolism genes (**d**). **e** Box plot and heatmap representing changes in the transcriptional activity of virus-associated carbon (CAZyme) metabolism genes linked with mesophilic MAGs (OGT < 50 °C). In all (**a**–**e**), the mean transcriptional activity (MAGs and vOTUs) shown in boxplots is based on transcript abundances (transcripts per million, TPM) normalized by MAG and vOTU abundances. Box plots encompass 25–75th percentiles, whiskers show the minimum and maximum values, and the midline shows the median (dots present the biologically independent samples, asterisks denote for significant differences (*p < 0.05, **p < 0.01. n.s, no significant differences). Heatmaps show the transcriptional activity (MAGs or vOTUs) based on non-normalized transcripts abundances (transcripts per million, TPM). In (**c** and **d**), selected CAZymes include GHs, GTs, PLs, CEs, CBMs, and AAs. Nitrogen metabolic pathways include assimilatory nitrate reduction, dissimilatory nitrate reduction, nitrification, and nitrogen fixation pathways. More detail about the functional genes included can be found in Supplementary Data 6 and 7, respectively.

To explore which bacteria and viruses were active during HTC, we mapped metatranscriptomic reads against metagenomic contigs, including assembled MAGs and vOTUs. Approximately, 74% of quality filtered mRNA reads could be mapped back to metagenomic assemblies. The transcriptional activity of bacterial MAGs followed closely different phases of HTC (Fig. 3a). Specifically, mesophilic bacteria were relatively more active at the initial phase, including MAGs belonging to *Armatimonadota* (34.2%) and *Actinobacteriota* (22.5%), while thermophilic bacteria became active during the hyperthermophilic phase (D15), including *Deinococcota* (74.0%) and *Firmicutes* (22.8%) (Fig. 3a and Fig. S8). Changes in bacterial community activity (based on RNA abundance matrix of MAGs) were positively correlated with several composting properties (Fig. S9). For example, the activity of mesophilic bacteria was highly correlated with carbon cycling (total carbon and inorganic carbon content) and OM degradation (Mantel’s *r* = 0.25–0.80, *p* < 0.05), while the activity of thermophiles was associated with composting temperature and nitrogen cycling (Mantel’s *r* = 0.25–0.60, *p* < 0.05).

In case of viruses, 98.5% of vOTUs were active closely following the composting temperature during HTC (Fig. 3b and Table S4). Of the taxonomically assigned vOTUs, *Autographiviridae* (45.5%) and *Vilmaviridae* (20.6%) were the most active viruses at the beginning of composting (D0, Fig. S10). While the activity of *Autographiviridae* reduced to 1.3%, the activity of *Matshushitaviridae* significantly increased to 90.2% during the hyperthermophilic phase (Fig. S10). Overall, mesophilic viruses were relatively more active at the beginning of composting and especially unknown virus NODE_4560_length_5650_cov_2.431093 had high transcriptional activity (3.5%) (Fig. 3b, Table S4). In contrast, thermophilic viruses were more active during hyperthermophilic (D15) and maturation (D27) phases, with unclassified virus NODE_636_length_12113_cov_2.763359 showing high transcriptional activity during the hyperthermophilic phase (Fig. 3b, Table S4). Similar patterns of activity were observed with viruses that could be classified only based on their predicted host taxa (Fig. S10). The activity of mesophilic viruses was only correlated with carbon cycling (TC and IC) and OM degradation (Mantel’s *r* = 0.5–0.8, *p* < 0.05, Fig. S9), while the activity of thermophilic viruses was significantly associated with composting temperature and carbon and nitrogen cycling (Mantel’s *r* = 0.25–0.50, *p* < 0.05, Fig. S9). Together, these results show that both bacterial and viral activity were associated with nutrient cycling during HTC.

### Viruses drive nutrient cycling via expression of auxiliary metabolic genes and through top-down regulation of bacterial abundances and activity

To understand the effects of viruses and bacteria on nutrient cycling at the functional level, we explored the expression dynamics of catabolic genes carried by both bacteria and viruses. All bacterial MAGs (227) encoded essential metabolism genes (Table S5). Specifically, carbohydrate-active enzymes (CAZymes; 117 and 89 genes per MAG on average for mesophiles and thermophiles, respectively), including glycoside hydrolases (GHs), glycosyl transferases (GTs), polysaccharide lyases (PLs), carbohydrate esterases (CEs), carbohydrate-binding modules (CBMs) and auxiliary activities (AAs), indicative of their importance in the decomposition of polysaccharides. Also, proteinase genes were detected in all MAGs (on average 52.1 and 46.3 genes per each mesophilic and thermophilic MAG, respectively), while 70.6% of MAGs (2.6 and 0.89 genes per each mesophilic and thermophilic MAG, respectively) harbored genes linked with denitrification (53.7%), dissimilatory nitrate reduction (44.4%) and nitrogen fixation (3.5%). The expression of most of these genes followed different phases of HTC (Fig. 3c–e). Specifically, carbohydrate metabolism genes (CAZymes, *F*_3.8_ = 37.7, *p* < 0.0001) were expressed by thermophiles during the thermophilic phases of HTC (D4 and D15, Fig. 3c), while nitrogen metabolism genes (*F*_3.8_ = 26.1, *p* = 0.00017) were expressed by mesophilic bacteria at the initial phase of composting (Fig. 3d). The transcriptional activity of CAZymes and nitrogen metabolic genes by thermophilic and mesophilic MAGs was positively associated with nutrient turnover during the HTC (Mantel test, *p* < 0.05, respectively).

We also compared the abundance and activity of virus-encoded auxiliary metabolic genes (AMGs) for the nutrient cycling. Around 4% of annotated viral ORFs were linked with carbohydrate transport and metabolism and amino acid transport and metabolism (Fig. S11). Viral AMGs identification was further examined using DRAM-v and manual curation. A total of 90 putative AMGs were found in 75 mesophilic phages (linked to mesophilic hosts), representing carbohydrate metabolism, amino acid metabolism and phosphorus metabolism KEGG categories (Table S6). A total of 34 putative AMGs belonged to 10 CAZyme families involved in breakdown of polysaccharide compounds, such as hemicellulose and chitin (Fig. 3e and Table S6). In addition, 14 AMGs were associated with peptidase and amino acid metabolism and these AMGs were present on 14 viral contigs. In addition, two putative AMGs involved in phosphorus metabolism (*e.g*., phosphate starvation-inducible protein *PhoH*, alkaline phosphatase D *phoD*) were detected on 10 viral contigs (Table S6). In support with previous analyses, no AMGs associated with inorganic nitrogen metabolism was found in any of the viral contigs. Almost all identified AMGs were expressed during the composting (99.5%), indicative of their importance in degradation of carbohydrates and exopolysaccharides (Fig. S12). While the expression level of total AMGs significantly decreased during the thermophilic phase (Fig. S13a, F_3,8_ = 7.7, *p* = 0.009), the activity of CAZyme significantly decreased (Fig. 3e, F_3,8_ = 9.4, *p* = 0.0053). As a result, viral CAZyme activity correlated positively with carbon cycling during HTC (*R*^2^ = 0.55, *p* < 0.0001), while no significant relationship with nitrogen cycling was observed (*R*^2^ = 0.14, *p* = 0.15, Fig. S13b). Together, these results suggest that AMGs encoded by mesophilic viruses contributed to degradation of complex carbohydrates during the non-thermophilic phase of composting.

**Fig. 4:**
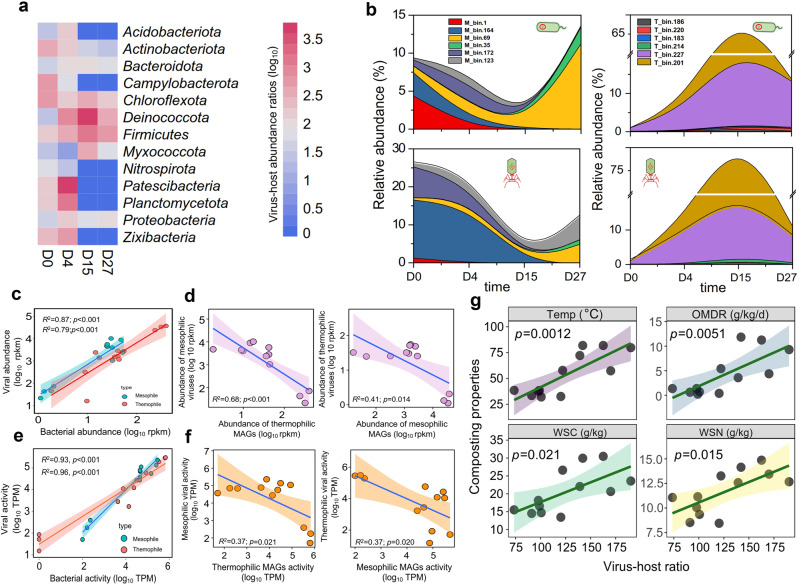
Lineage-specific virus–host abundances and activity are coupled during HTC. **a** Heatmap showing changes in the mean virus-host abundance ratios (VHRs) based on all MAGs and vOTUs grouped by predicted host bacterial taxonomy over four sampling time points (D0, D4, D15, D27) during HTC. **b** Changes in the relative abundance of dominant mesophilic (left panel) and thermophilic (right panel) MAGs (upper panel) and their associated viruses (lower panels; vOTUs) during HTC. Linked MAGs and vOTUs are shown with same colors. **c** Positive relationships between dominant mesophilic and thermophilic MAGs and their viruses based on relative abundances. **d** Negative relationships between the relative abundances of dominant mesophilic and thermophilic viruses with mesophilic and thermophilic MAGs, respectively. **e** Positive relationships between dominant mesophilic and thermophilic MAGs and their viruses based on metatranscriptomics data (transcriptional activity based on non-normalized transcripts per million reads (TPM)). **f** Negative relationships between the relative activity of dominant mesophilic and thermophilic viruses with mesophilic and thermophilic MAGs, respectively. **g** Significant positive correlations between total VHRs and changes in composting properties during HTC. Shaded area shows 95% confidence interval around the fitted mean line. In all panels (except for **a** and **b**), data shows mean values of three biological replicates per treatment (*n* = 3).

We investigated if viruses could have affected bacterial catabolic activity via top-down density regulation of their hosts based on the lineage-specific virus-host ratios (VHR; estimated using MAG and vOTU data). Overall, viral abundances clearly surpassed bacterial abundances, and the mean VHR of all virus-host lineages showed a clear increase at thermophilic phases (ranging from 73.6 to 190.1; F_3,8_ = 9.0, *p* = 0.006, Fig. S14a). The highest VHRs were observed with *Deinococcota* at D15, followed by *Firmicutes* at D27, and the dynamics of VHRs followed changes in the composting temperature (Fig. 4a). Twelve most dominant mesophilic and thermophilic MAGs (six each), whose abundances accounted for 9.1–69.7% of all MAGs during HTC, were chosen to study the top-down regulation of bacterial densities by their viruses (Fig. 4b). The relative bacterial and viral abundances were tightly coupled (*R*^2^ = 0.79 and *R*^2^ = 0.87, *p* < 0.001) during HTC (Fig. 4c), while both the activity (*R*^2^ = 0.93 and *R*^2^ = 0.96, *p* < 0.001) and abundances (*R*^2^ = 0.79 and *R*^2^ = 0.87, *p* < 0.001) of thermophilic and mesophilic MAGs correlated positively with their viral abundances and activities (Fig. 4e). However, the relative abundances and activity of mesophilic and thermophilic bacteria and viruses correlated negatively with each other, showing a clear microbial succession during HTC (Fig. 4d–f). As a result, the mean VHR of thermophilic MAGs increased with composting temperature (specifically for the *Thermus thermophilus*, T_bin.227, D15; F_3,8_ = 5.7, *p* = 0.022), while the VHRs of mesophilic MAGs were clearly the highest during non-thermophilic phases (D0 and D27; F_3,8_ = 5.5, *p* = 0.023, Fig. S14b). Moreover, changes in VHRs correlated positively with composting temperature (*R*^2^ = 0.63, *p* = 0.0012), WSC (*R*^2^ = 0.31, *p* = 0.034), WSN (*R*^2^ = 0.41, *p* = 0.015) and OM degradation rate (*R*^2^ = 0.56, *p* = 0.0056, Fig. 4g), suggesting that viruses boosted nutrient cycling via top-down density regulation of host bacteria during HTC.

**Fig. 5:**
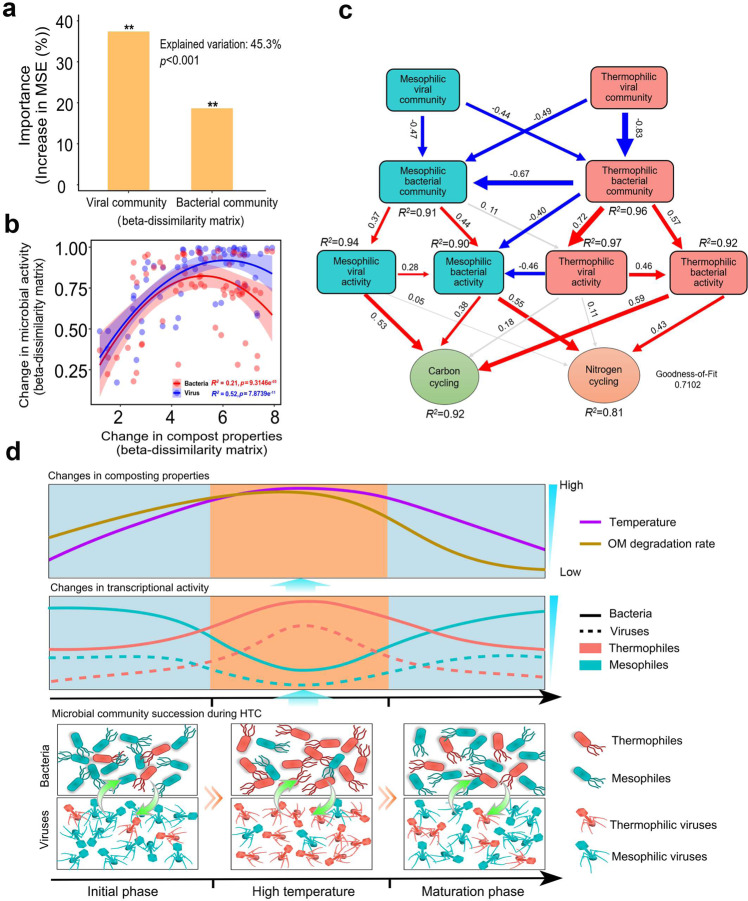
Comparison of the relative importance of viral and bacterial communities for the nutrient cycling during HTC. **a** Percentage increases in the MSE (mean squared error) estimating the importance of viral (vOTU) and bacterial (MAG) communities in explaining variation in nutrient cycling during HTC (higher MSE% values imply increased importance of a given predictor). Random forest mean predictor importance measure was computed for each tree and averaged over the forest (5000 trees). **b** Significant non-linear correlations between bacterial (black line) and viral (blue line) community activity with nutrient turnover (based on all biogeochemical parameters) during HTC based on metatranscriptomic datasets. **c** Partial least-squares path model (PLS-PM) comparing the relative importance of different factors explaining nutrient cycling during HTC. PLS-PM describes the relationships between viral and bacterial communities (beta-dissimilarity based on mesophilic and thermophilic MAGs or vOTU), viral and MAG catabolic activity. Strengths of path coefficients are shown as arrow width and numbers beside them, while blue, red and gray colors indicate negative, positive, and non-significant effects, respectively. Path coefficients and coefficients of determination (*R*^2^) were calculated after 999 bootstraps and significance levels are indicated when *p* < 0.05. In (**b**), gray cloud represents a 95% confidence interval around the predicted values. **d** Schematic illustration describing the relationships between mesophilic and thermophilic bacteria and their viruses in relation to nutrient cycling during HTC.

Multiple regression models were constructed to explain changes in nutrient cycling (comparison of dissimilarity matrices based on all composting properties in time) with relative bacterial and viral community activity (changes in dissimilarity matrices in time based on metatranscriptomes). The activity of bacterial and viral communities (based on MAGs and vOTUs) explained 45.3% of the total variance in nutrient turnover. Viruses showed a relatively greater contribution compared to bacteria (Fig. 5a), which was especially clear with carbon cycling, while bacterial activity was more strongly associated with nitrogen cycling (Fig. S15). The activity of MAGs (*R*^2^ = 0.21, *p* < 0.001) and vOTUs (*R*^2^ = 0.52, *p* < 0.001) correlated positively with changes in nutrient cycling (Fig. 5b), suggesting that nutrient turnover and bacterial and viral activities were coupled during HTC. To further disentangle the relative importance of mesophilic and thermophilic bacteria and viruses on carbon and nitrogen cycling, partial least-squares path models (PLS-PM) were constructed. We found that bacterial community composition was positively associated with both bacterial and viral activity (Fig. 5c), while thermophilic bacteria had negative association with mesophilic bacteria as predicted by the observed microbial community succession during HTC. Moreover, both mesophilic and thermophilic viruses were positively associated with their host bacteria, indicative of tight coupling of virus-bacteria dynamics during HTC (Fig. 5c). In line with previous analyses, mesophilic and thermophilic bacteria were positively associated with both carbon and nitrogen cycling, with mesophiles showing relatively stronger effects with nitrogen and thermophiles stronger effects with carbon (Fig. 5c). In contrast, only mesophilic viruses were associated with carbon cycling, while thermophilic viruses had no significant associations with nutrient turnover. Together, these findings demonstrate that both mesophilic and thermophilic viruses drove nutrient cycling by regulating bacterial biomass and activity during HTC.

### RNA viruses were not associated with nutrient cycling during HTC

As recent studies have suggested that RNA viruses could also be important but overlooked players underlying the ecosystem functioning [77–80], we used the RNA-dependent RNA polymerase (RdRP) hallmark gene as the target gene to study of the diversity and composition of RNA viruses during composting. Approximately, 0.23% of quality filtered mRNA reads could be mapped back to these RNA viral contigs (Fig. S16a), suggesting that RNA viruses had a very low frequency in the metatranscriptome. Of these 86 potential RNA viruses, 26 could be clustered with taxonomically known viruses in viral RefSeq database (v216). Primarily, they belonged to four phyla: *Kitrinoviricota* (69.2%), *Negarnaviricota* (11.5%), *Pisuviricota* (11.5%), and *Lenarviricota* (7.6%) (Fig. S16b). More than 50% of viruses belonged to *Virgaviridae* family that mainly consists of plant viruses, and only two viruses belonged to *Fiersviridae* family, which have been associated with bacteria (Fig. S16c) [87]. Most of these RNA viruses (83%) were present at the initial and maturation phases of composting, and only a few RNA phages (17%) were detected in samples collected during thermophilic phase (D4 and D15). This may be due to sensitivity of RNA viruses to heat [88]. In support of this, abundances of RNA viruses (based on mapping RNA sequencing reads back to RdRP-bearing contigs using coverM (v0.61, https://github.com/wwood/CoverM) followed closely different phases of composting (Fig. S16d, e), with most RNA viruses having high abundances at initial and maturation phases of composting, while almost vanishing during thermophilic phase. We also analyzed the presence of dsDNA phages in metatranscriptomic dataset using the same methods as with metagenomics (except for VIBRANT tool). A total of 68 putative dsDNA phage contigs (with sizes >5 kb) were obtained from the metatranscriptomic assemblies. After clustering (95% nucleotide similarity and over 85% coverage), a total of 41 dsDNA viruses were retained. By comparing the dsDNA viruses contigs derived from transcriptomic data and metagenomic data, 89% viruses (61 of 68) could be assembled from both transcriptomic and metagenomic data, suggesting that very few dsDNA viruses exclusively exist in metatranscriptomic dataset. As a result, the RNA viral community abundance (based on beta-dissimilarity of abundance matrix) did not correlate with changes in composting properties (Mantel statistic *r* = 0.0173, *p* = 0.35), which suggests that they did not contribute to the nutrient cycling during composting.

## Discussion

Here we studied the role of viruses in nutrient cycling during hyperthermophilic composting using replicated and temporally sampled metagenomics and metatranscriptomics datasets. We found that bacterial and viral community dynamics were tightly coupled and followed different phases of HTC and degradation of organic matter. Specifically, while mesophilic viruses participated in nutrient cycling by encoding AMGs linked with carbon cycling, thermophilic viruses played only an indirect role via top-down regulation of thermophilic bacterial densities. Nutrient turnover correlated positively with virus–host ratio, which suggest that relative viral abundances could be used as an indicator of ecosystem functioning (Fig. 5d). These effects were driven by DNA viruses as RNA viruses were mainly associated with eukaryotes and were not correlated with nutrient cycling during composting. Our results are in line with previous studies linking DNA viruses to nutrient turnover in terrestrial ecosystems [4, 10, 89], highlighting the role of viral diversity and activity for terrestrial biogeochemical cycles.

Both bacterial and viral communities followed closely different phases of composting, with mesophiles and thermophiles dominating non-thermophilic and thermophilic phases, respectively. Moreover, viruses showed high activity even during the hyperthermophilic phase (>90 °C) and consistently surpassed bacterial abundances in terms of virus-host abundance ratio. Mesophilic and thermophilic bacteria and their viruses showed clear microbial community succession, where the initial phase of composting was dominated by mesophiles, which were subsequently replaced by thermophiles and subsequently by mesophiles towards the end of the HTC. Although similar compositional succession of bacterial and fungal communities have been observed in previous composting experiments [19, 90], this is the first evidence demonstrating that viruses can also drive ecological succession in microbial communities during HTC. These findings are also indicative of “Kill-the-Winner” hypothesis, where viruses target and regulate the most abundant group of host bacteria, reducing the dominance effects and evening out competition between different bacterial taxa [4, 89]. Such dynamics could explain the observed community shift between thermophilic and maturation phases of HTC, where thermophilic viruses likely drove down the abundances of thermophilic bacteria, giving rise to mesophilic bacteria and their phages. For example, *Thermus* and *Planifilum* bacterial genera play important role in heat production during HTC [17] and several lytic phages that infected *Thermus thermophilus* (T_bin.227) and *Planifilum fulgidum* (T_bin.201) were identified, including five potentially novel *Thermus* viruses that had genome sizes about 5 kbp similar to hyperthermophilic phage φOH3 isolated from Obama hot spring [91]. While 61% of detected phages were predicted to be lytic, it is possible that some of the correlations between bacterial and viral taxa were also driven by lysogenic phages or prophages because unfiltered DNA samples were used for metagenomics. As a result, our dataset likely underestimates phage diversity, and phage enrichment [92] should be used in future studies. Moreover, future work should also consider the potential role of RNA viruses for HTC, which we did not explore in detail as compost-associated bacteria are most often associated with DNA viruses [19]. Nevertheless, our results suggest that a small portion of thermophilic viruses played a key role in microbial activity during the thermophilic phase of HTC, indicating that compost ecosystem functioning was at least temporally driven by low-diversity microbial communities. Terrestrial phages could hence be important drivers of biogeochemical cycling in soil ecosystems via “viral shunt” akin to marine phages [11, 93].

Viral and bacterial activities were also positively correlated with each other, and virus–host ratio correlated positively with changes in both carbon and nitrogen cycling. While both mesophilic and thermophilic bacteria actively expressed genes linked with carbon and nitrogen cycling, only mesophilic viruses encoded carbon metabolism-related AMGs, while no metabolism-related AMGs were found in thermophilic viruses. These findings are in line with previous studies conducted in marine and soil ecosystems, which have identified a variety of AMGs linked to nitrogen metabolism [94], carbon metabolism [95], phosphate metabolism [96], and the sulfur cycle [97] in mesophilic viruses. However, while carbon-linked viral AMGs are frequently detected in various environments [4, 14, 27], viral AMGs associated with nitrogen cycling have been observed less often [94] and were not either found in our study. All carbon-associated viral AMGs were carbohydrate-active enzymes (CAZymes) and their transcriptional activity was significantly correlated with carbon turnover during HTC. One potential explanation for the relatively higher prevalence of carbon-associated viral AMGs is that they might provide more benefits for the host bacteria as composting matrix contains lots of organic carbon [16, 98]. No CAZymes were encoded by thermophilic viruses. One reason for this could be that thermophilic viruses have adapted to encode non-metabolism-related AMGs to improve bacterial and their own survival in stressful environments [3, 14]. In support of this, many thermophilic AMGs were associated with amino acid and nucleotide metabolism, and their transcriptional activity was significantly increased during the thermophilic phase. Despite disparate effects on metabolism, both types of viruses played indirect role in nutrient cycling by regulating bacterial abundances and catabolic activity through top-down density control. However, more work is required to directly validate the functioning of discovered viral AMGs in the future. Also, as only a very small fraction of vOTUs were of high quality, our analysis likely underestimated the functional diversity and gene content of viruses. Finally, we used our metatranscriptomics dataset to explore the potential role of RNA viruses in composting. Most of the detected RNA viruses (82%) were associated with eukaryotic hosts and had low relative abundances during hyperthermophilic phase of composting. This is in line with previous studies suggesting that RNA viruses have mainly eukaryotic hosts [77] and that nutrient cycling during composting is mainly driven by prokaryotic organisms [19, 21]. RNA viruses therefore likely played a small role in nutrient cycling during HTC.

In conclusion, this study provides evidence for the importance of viruses in terrestrial biochemical cycles and nutrient turnover, especially in thermophilic extreme environments. Much of the discovered viral diversity was absent in reference sequence databases, highlighting the continued discovery of novel viral diversity and its role in microbial ecosystems. One reason for this could be that HTC environment is highly specific to bacterial and viral taxa that are not found in other terrestrial or aquatic ecosystems. In addition to exerting strong top-down regulation and driving microbial community succession between mesophilic and thermophilic bacteria, viral AMGs were associated with carbon cycling during the mesophilic phases of composting. Further, viral abundances often surpassed bacterial abundances and high virus-host ratio peaks were associated with efficient degradation of organic matter. Relative viral abundances could hence potentially be used as an indicator of efficient nutrient cycling and microbial ecosystem functioning to optimize productivity of biotechnological and agricultural systems.

## Supplementary information

supplemental materials

Dataset 1 to 8

## Data Availability

Raw reads data generated from both amplicon and shotgun sequencing in this study have been deposited with the NCBI SRA (PRJNA861164, PRJNA861429, and PRJNA861433) and are publicly available. Short Reads Archive accession numbers for individual reads are listed in Supplementary Data 7. All of assembled viral and bacterial genomes and R codes used in this study are available at Zenodo (https://zenodo.org/record/7397132#). The authors declare that the other main data supporting the findings of this study are available within this Article and in the Supplementary Information files.
