# Supplementary material for: Mesophilic and thermophilic viruses are associated with nutrient cycling during hyperthermophilic composting
Source: ISME J. 2023 Apr 8;17(6):916–30. doi: 10.1038/s41396-023-01404-1 (PMC10202948; doi:10.1038/s41396-023-01404-1)
Supplement: Supplementary file 1 — supplemental materials [file 41396_2023_1404_MOESM1_ESM.pdf]

Supplementary Information

Mesophilic and thermophilic viruses are associated with nutrient cycling during hyperthermophilic composting

Hanpeng Liao[1,2], Chen Liu[1], Chaofan Ai[1], Tian Gao[1], Qiue Yang[1], Zhen Yu[3], Shaoming Gao[4], Shungui Zhou[1,2*], Ville-Petri Friman[5,6*]

Affiliations

[1] Fujian Provincial Key Laboratory of Soil Environmental Health and Regulation, College of Resources and Environment, Fujian Agriculture and Forestry University, Fuzhou 350002, China.

[2] Guangdong Laboratory for Lingnan Modern Agriculture, Guangzhou, 510642, China.

[3] Institute of Eco-Environmental and Soil Sciences, Guangdong Academy of Sciences, Guangzhou, 510650, China.

[4] School of Life Sciences, Sun Yat-sen University, Guangzhou 510275, China

[5] Department of Biology, University of York, Wentworth Way, YO10 5DD, York, UK

[6] Department of Microbiology, University of Helsinki, Helsinki, 00014, Finland

[*]Corresponding authors

Correspondence to Shungui Zhou (sgzhou@fafu.edu.cn) and Ville-Petri Friman (ville-petri.friman@helsinki.fi)

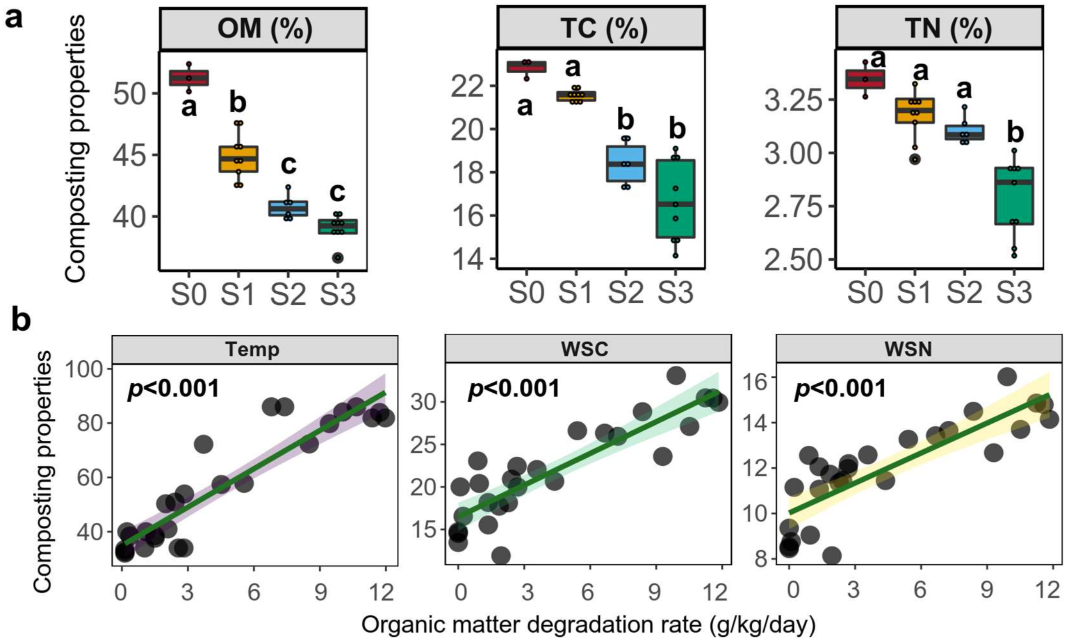

Fig. S1 Changes in composting properties during HTC. **(a)** Percentage change in organic matter (OM), total carbon (TC) and total nitrogen (TN) content during HTC. S0: initial raw material (D0); S1: hyperthermophilic stage (D4 to D9); S2: thermophilic stage (D15 to D21); S3: maturity stage (D27 to D45); data show mean ± SD of three biological replicates per treatment (n=3). Box plots encompass the 25–75th percentiles, the whiskers show the minimum and maximum values, and the midline indicates the median (n=3 biologically independent replicates). The different lowercase letters between treatments denote for significant differences at $p < 0.05$. **(b)** Positive correlations between composting properties and organic matter degradation rate. Temp: temperature; WSC: water soluble carbon; WSN: water soluble nitrogen.

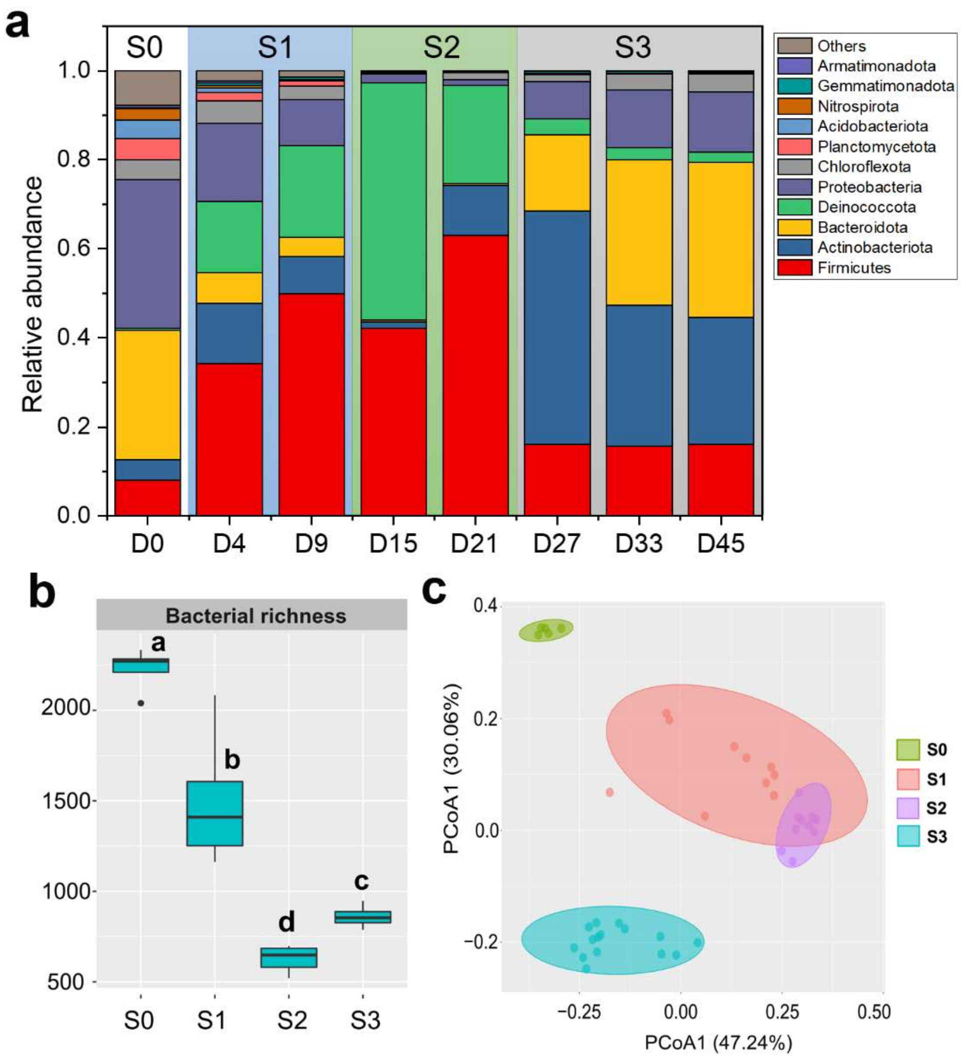

Fig. S2 Changes in bacterial community diversity and composition during HTC based on 16S rRNA amplicon sequencing. **(a)** Changes in bacterial composition at phylum level during HTC. S0: initial raw material (D0); S1: hyperthermophilic stage (D4 to D9); S2: thermophilic stage (D15 to D21); S3: maturation stage (D27 to D45). **(b)** Changes in bacterial richness during HTC. S0: initial raw material (D0); S1: hyperthermophilic stage (D4 to D9); S2: thermophilic stage (D15 to D21); S3: maturation stage (D27 to D45). Box plots encompass the 25–75th percentiles, the whiskers show the minimum and maximum values, and the midline indicates the median (n=3 biologically independent replicates). The different lowercase letters between treatments denote for significant differences at $p < 0.05$. **(c)** Comparison of bacterial community composition based on distance-based (weighted Unifrac) PCoA analysis during HTC.

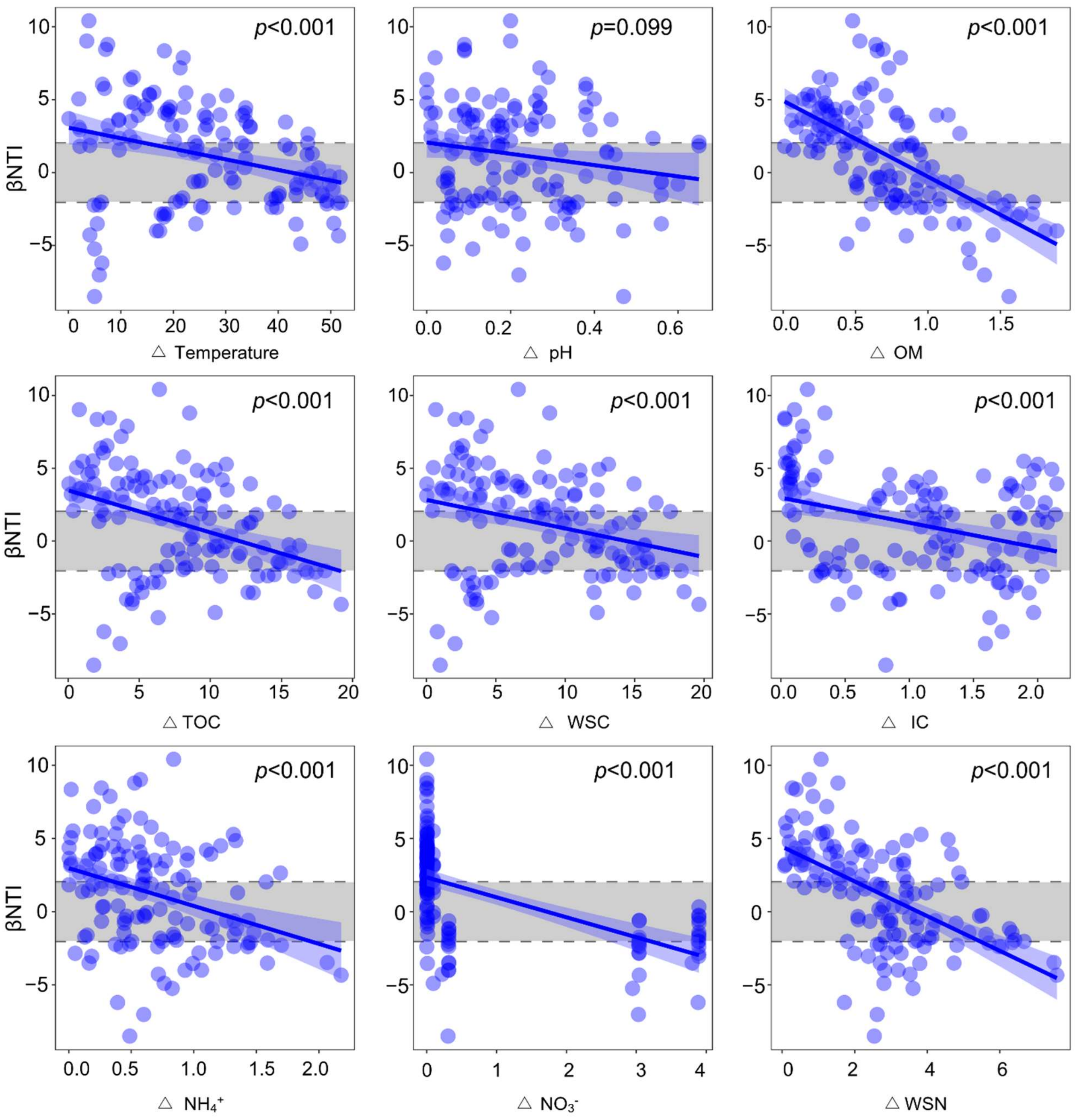

Fig. S3 The relationship between β-nearest taxon index (βNTI) and difference in composting properties composting during HTC. Blue circles represent pairs of samples where deterministic processes dominated community assembly (βNTI <-2: homogeneous selection, βNTI > +2: variable selection). The results of linear regression analysis in all panels are shown as blue color-coded best-fit trendlines for each point (the shaded areas depict the 95% confidence interval and R shows the slope of the best-fit trendlines). The abbreviations of composting properties are following: organic matter content (OM); total organic carbon content (TOC); water soluble carbon (WSC); inorganic carbon content (IC); ammonium (NH_4^{+}) and nitrate (NO_3^{-}) concentrations; water-soluble nitrogen (WSN).

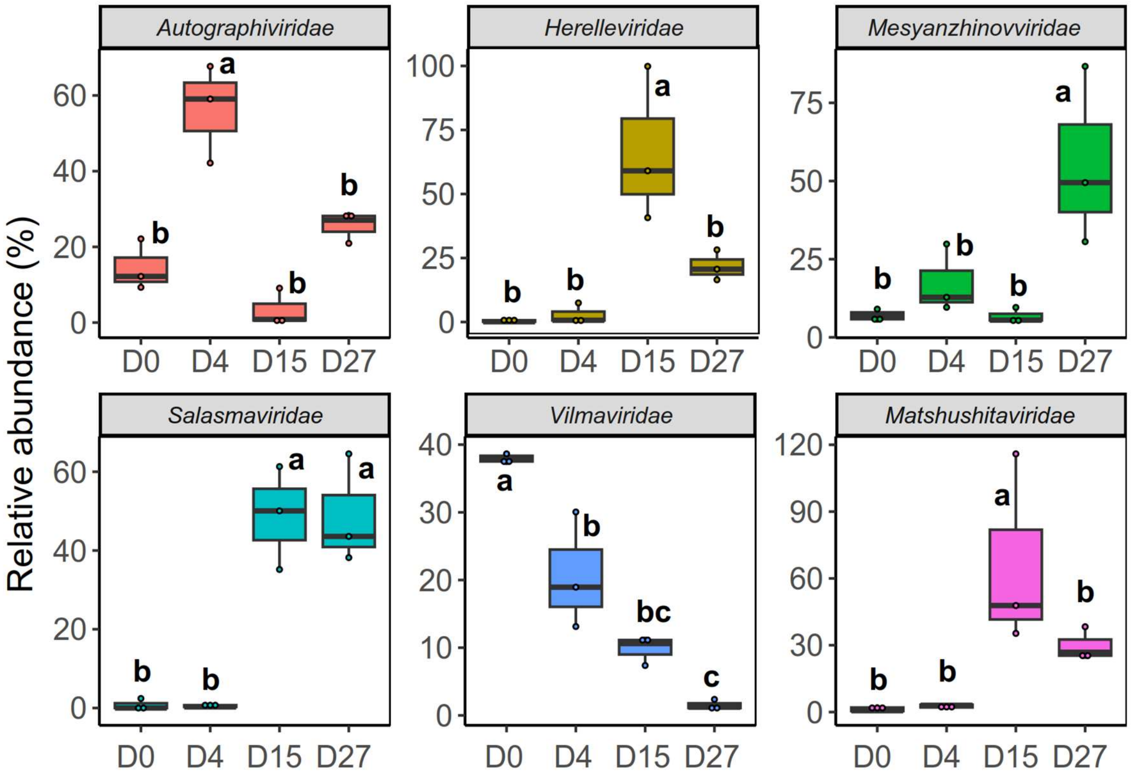

Fig. S4 Changes in relative abundances of taxonomically assigned viruses at family level during HTC (D0, D4, D15, and D27). Box plots encompass the 25–75th percentiles, the whiskers show the minimum and maximum values, and the midline indicates the median Data show mean ± SD of three biological replicates per treatment (n=3); different lowercase letters between treatments denotes for significant differences at $p < 0.05$.

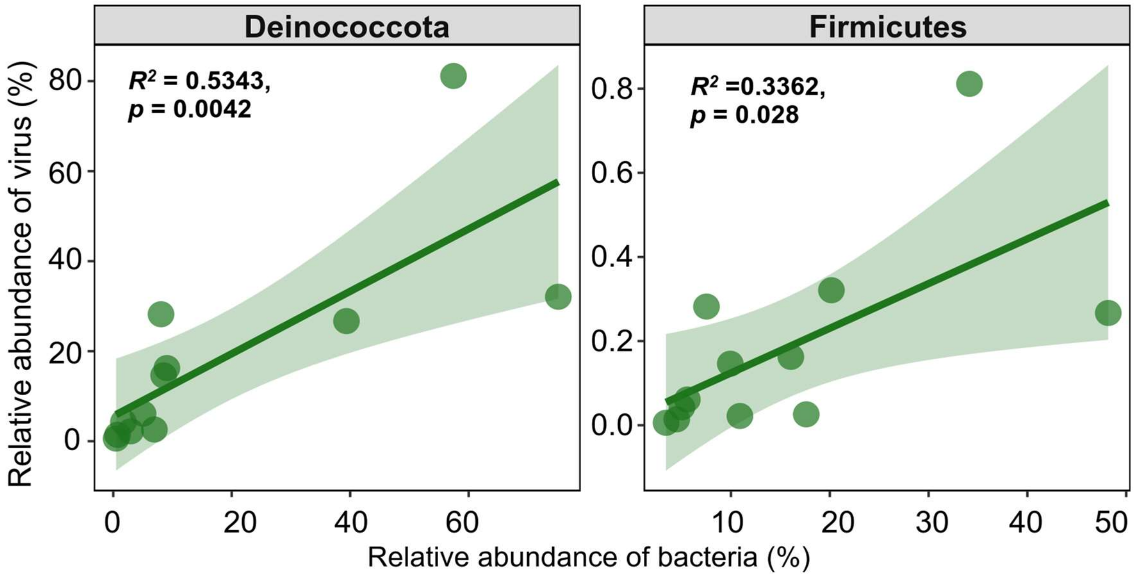

Fig. S5 The relationships between relative viral and their host bacterial abundances for *Deinococcota* (left) and *Firmicutes* (right) during HTC. The linear regression analysis in both panels shows best-fit trendline (the shaded areas depict the 95% confidence interval and adjusted R^2 the slope of the best-fit trendline).

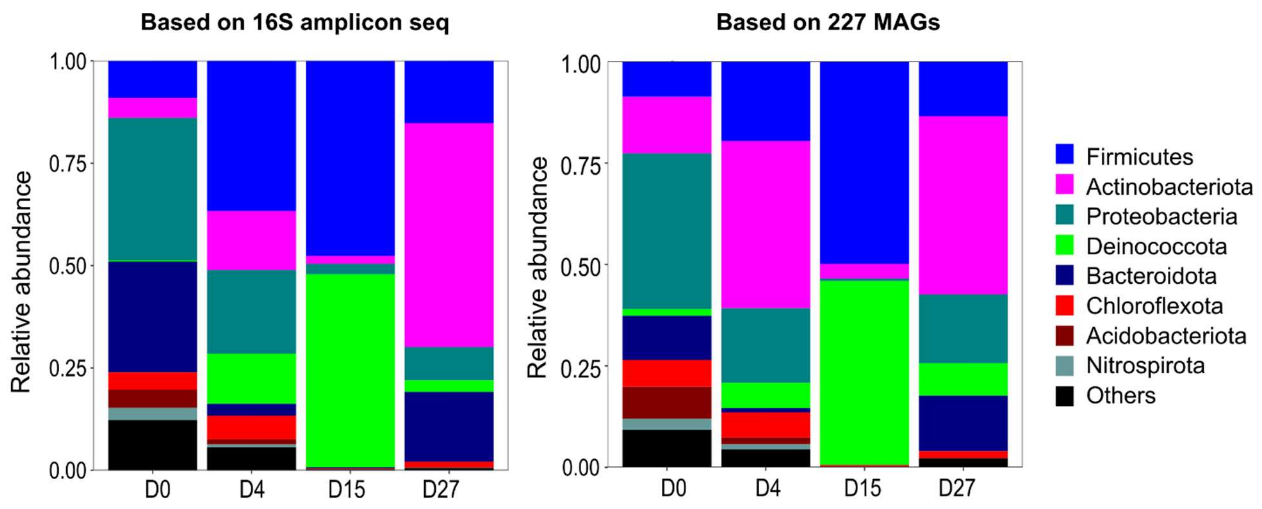

Fig. S6 Comparison of taxonomic composition and relative bacterial abundances at phylum level based on 16S rRNA gene amplicon sequencing (left) and 227 MAGs (right) at four sampling point (D0, D4, D15, and D27) during HTC.

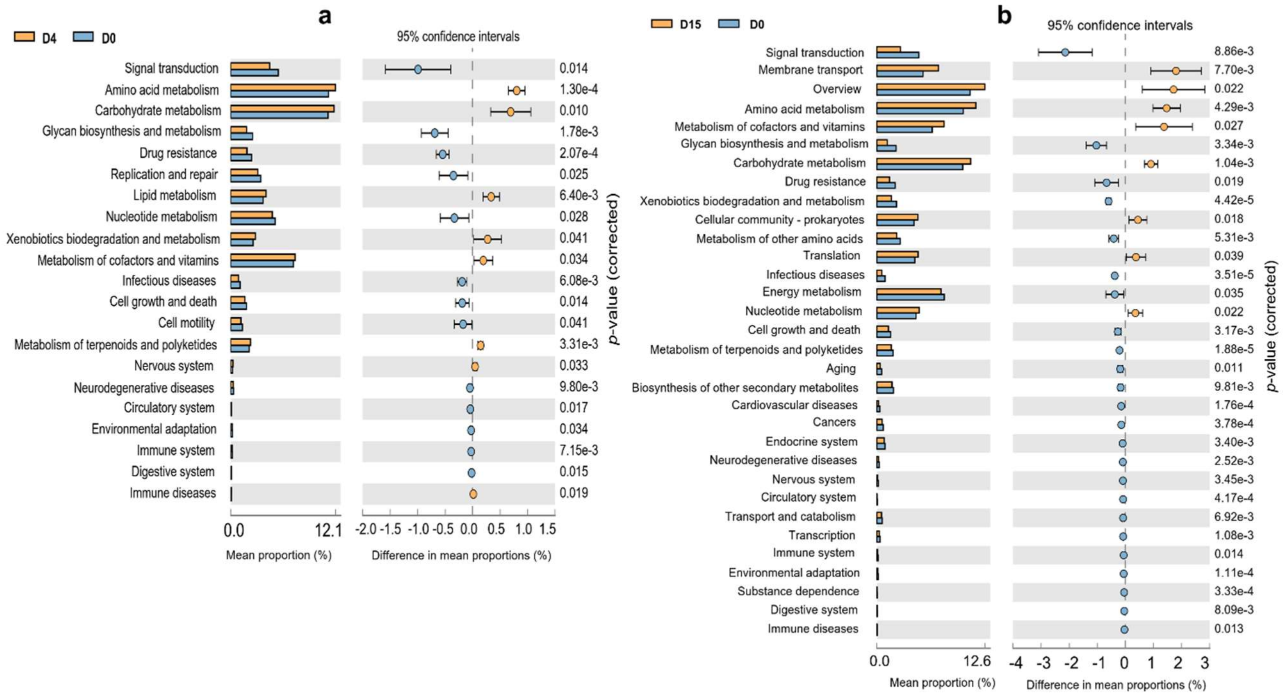

Fig. S7 Comparison of relative functional gene abundances between bacterial communities in initial composting raw materials (D0) and thermophilic phases (D4 and D15) during HTC.

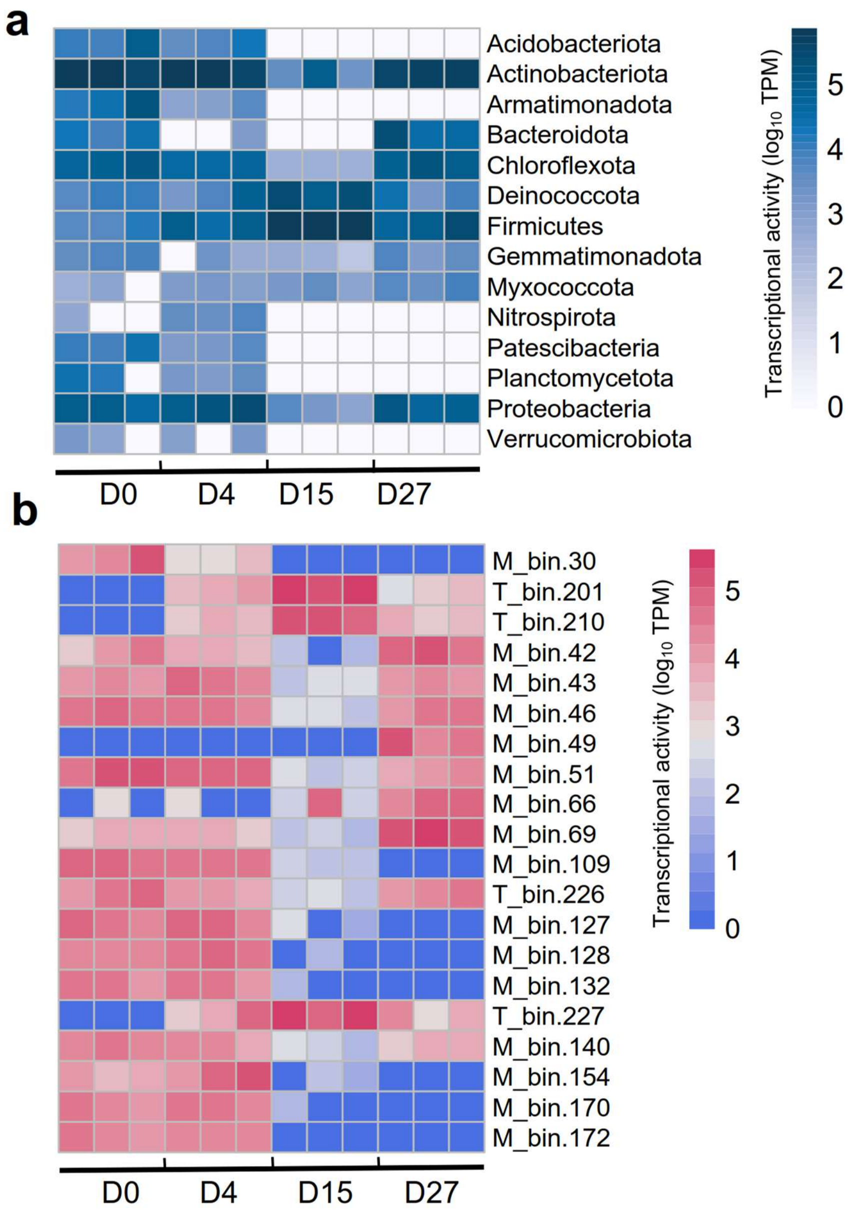

Fig. S8 Changes in transcriptional activity of bacterial MAGs at phylum level **(a)** and individual MAGs (top 20 most abundant) **(b)** at four sampling point (D0, D4, D15, and D27) during HTC.

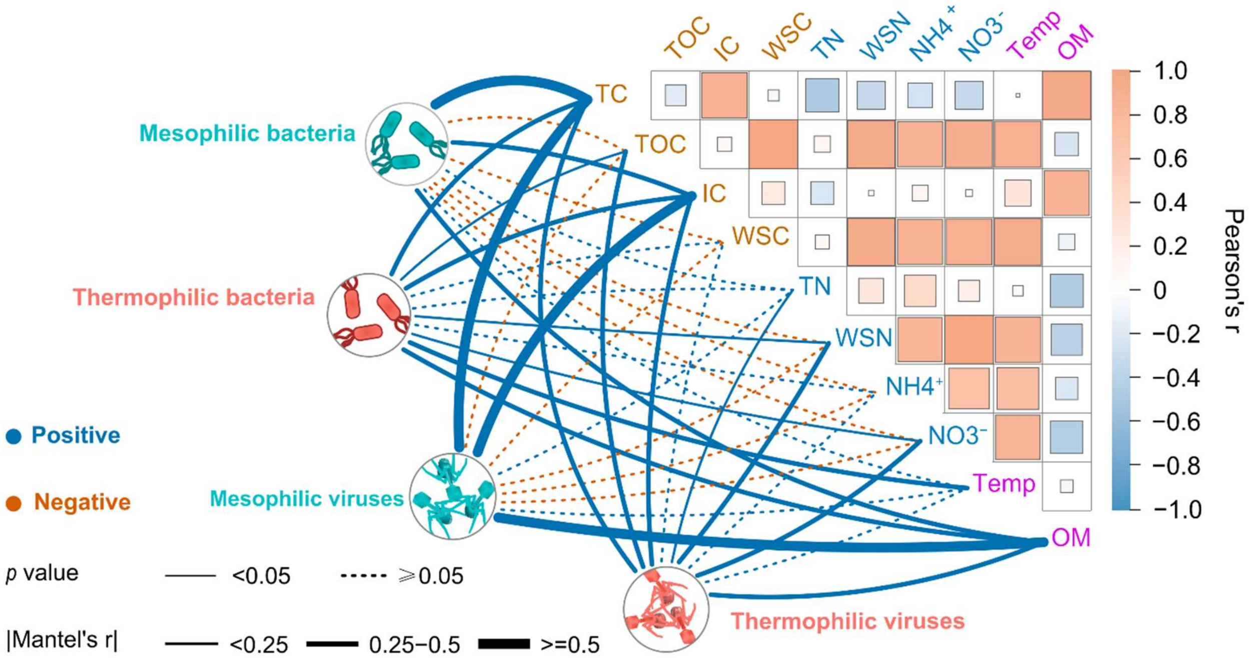

Fig. S9 Correlations between composting properties and microbial community activity based on transcriptional activity of mesophilic and thermophilic bacteria and viruses. Edge widths correspond to the absolute value of the correlation coefficient determined by the Mantel's statistics. Colors indicate correlation types. Solid and dashed lines denote for significant and non-significant correlations, respectively. Pairwise comparisons of environmental factors are shown in the matrix, with color gradient denoting Pearson's correlation coefficient. The abbreviations of composting properties are following: total carbon content (TC); total organic carbon content (TOC); inorganic carbon content (IC); water soluble carbon (WSC); total carbon content (TN); ammonium ($NH_4^{+)}$ and nitrate (NO_3^-) concentrations; water-soluble nitrogen (WSN); temperature (Temp); organic matter content (OM).

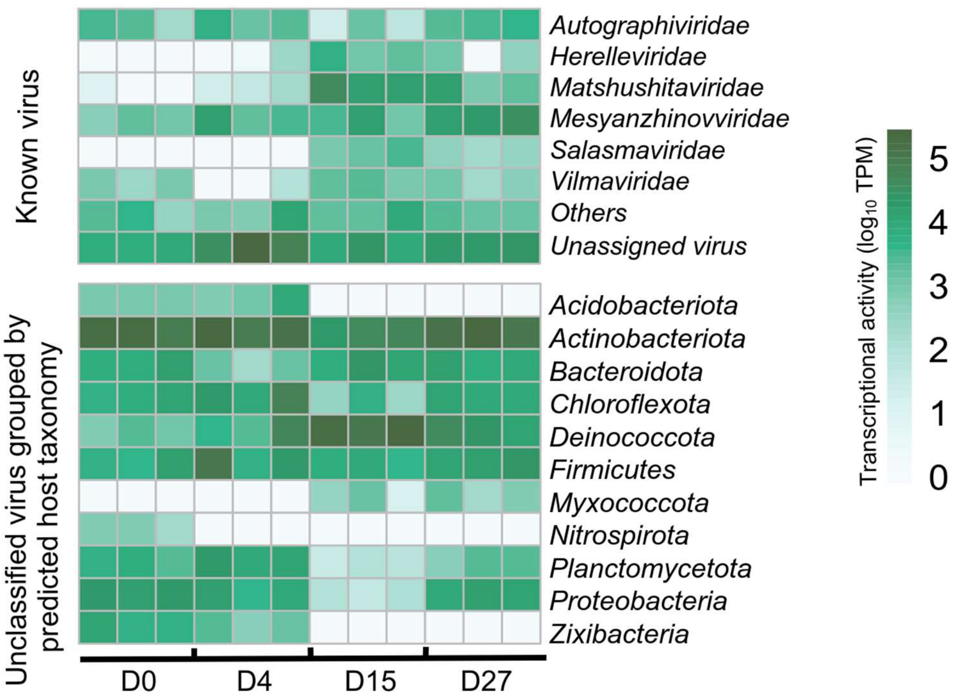

Fig. S10 Changes in viral transcriptional activity (vOTUs) averaged over taxonomic groups at four sampling point (D0, D4, D15, and D27) during the HTC.

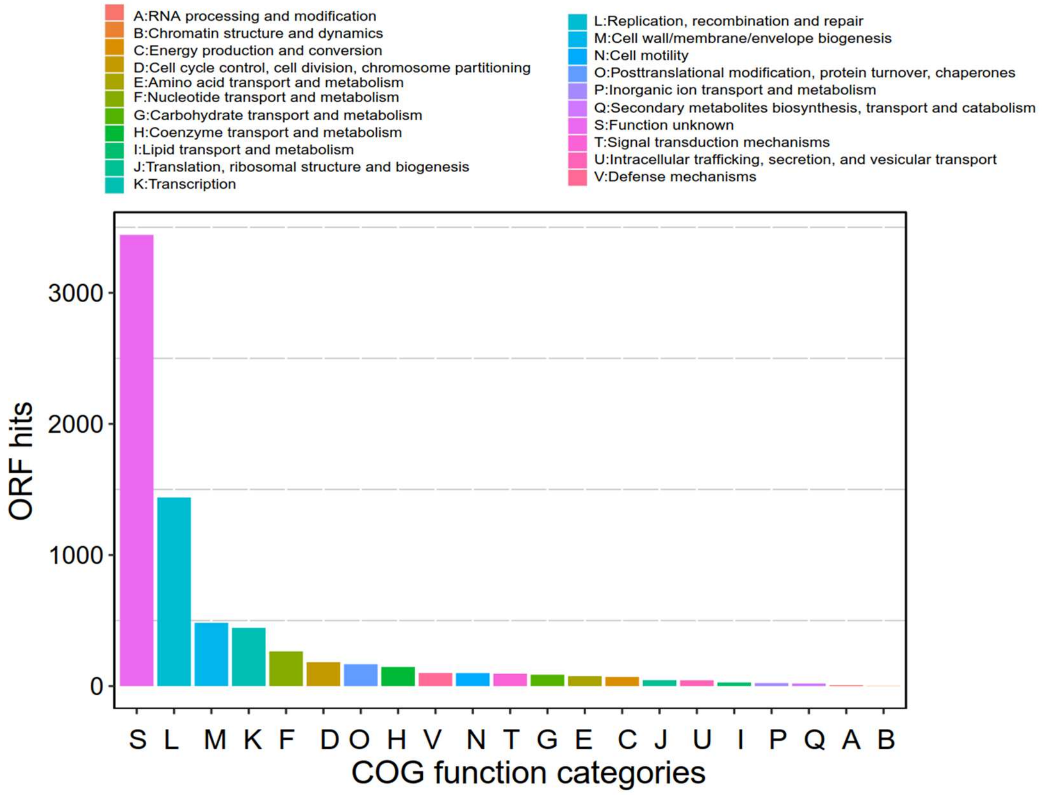

Fig. S11 The overall gene profile of all viruses summarized as viral ORF hits with eggNOG database.

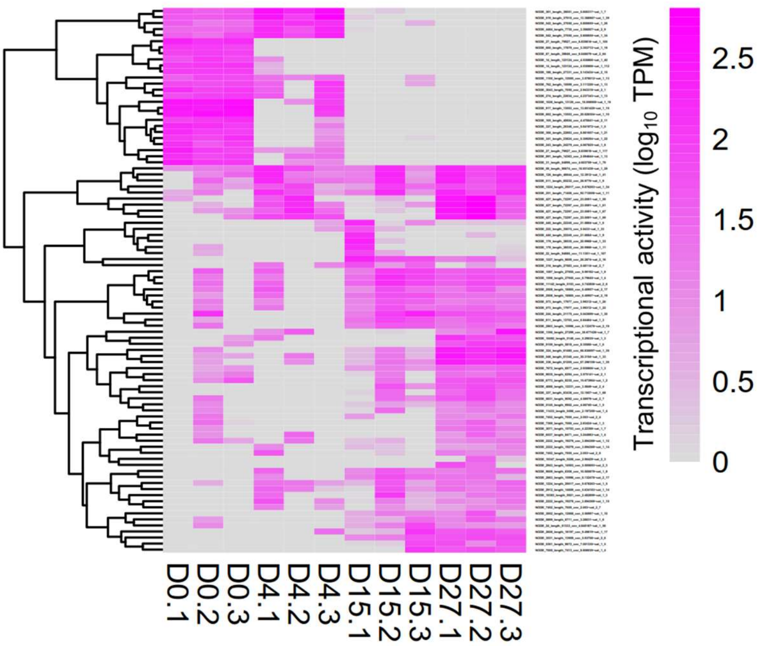

Fig. S12 Changes in transcriptional activity of individual viral AMGs at four sampling point (D0, D4, D15, and D27) during HTC.

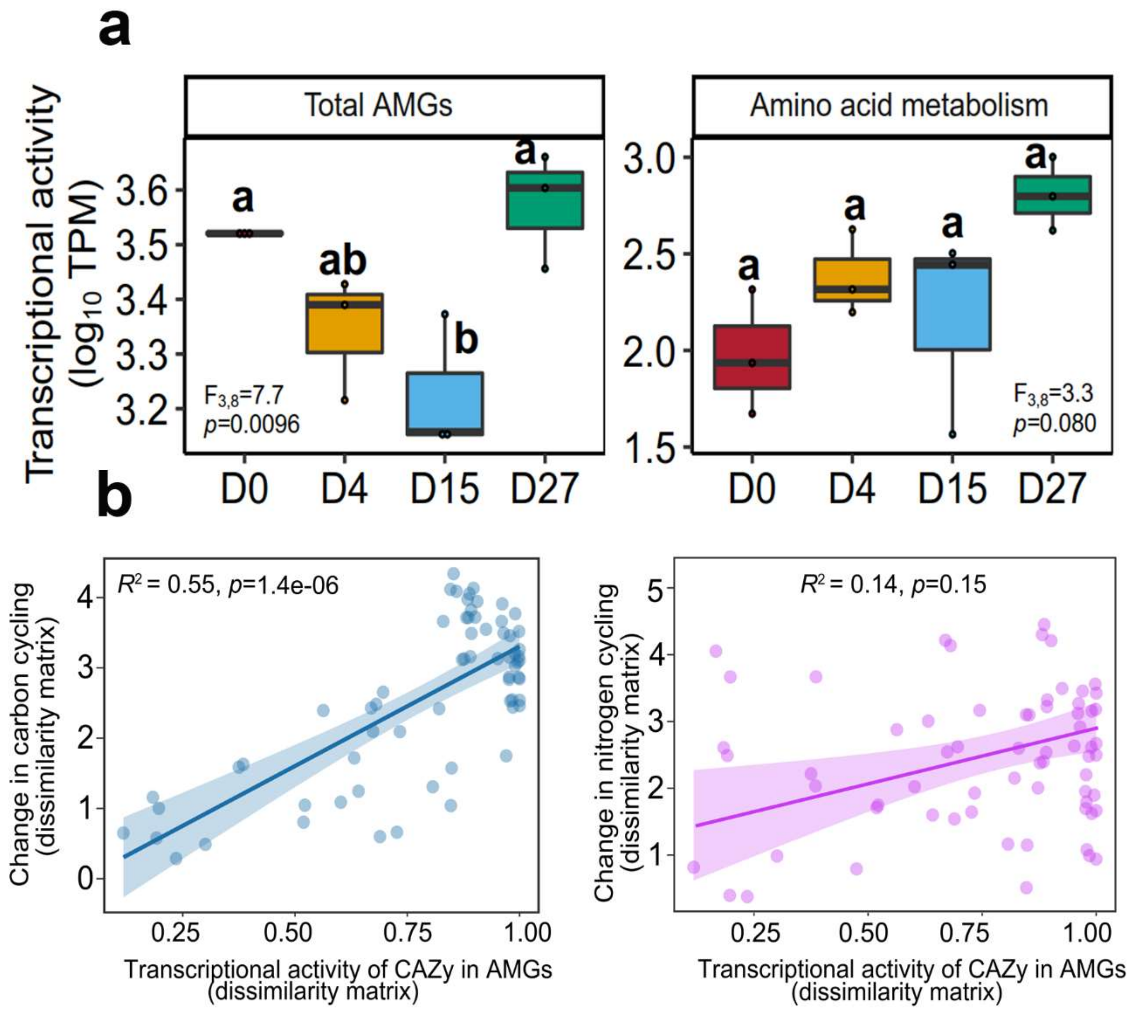

Fig. S13 Changes in total transcriptional activity of viral AMGs **(a)** associated with nucleotide metabolism (left panel) and amino acid metabolism (right panel) at four sampling point (D0, D4, D15, and D27) during HTC. **(b)** Correlations between transcriptional activity linked to CAZymes (based on dissimilarity distance) and carbon (left panel), and nitrogen (right panel) cycling during HTC. The carbon cycling index was based on change in dissimilarity matrix comprised of TC, IC, WSC, and TOC. The nitrogen cycling index was based on change in dissimilarity matrix comprised of TN, WSN, NH_4^+, and NO_3^-. In panel **(a)**, data show mean ± SD with of three biological replicates per treatment (n=3). In panel **(b)**, grey cloud represents 95% confidence interval around the predicted values. The different lowercase between treatments denotes for significant differences at $p < 0.05$.

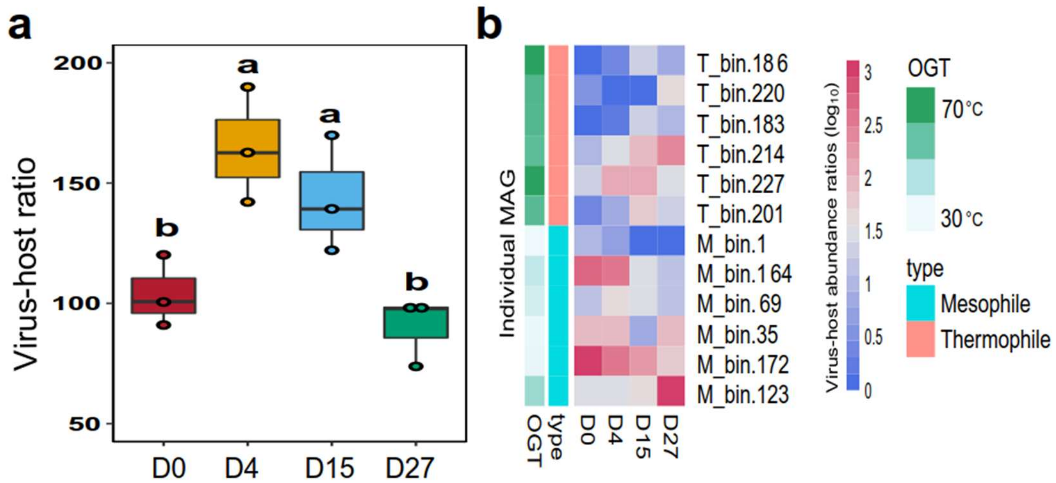

Fig. S14 Changes in mean virus-host ratios based on all (a) and representative (b) virus-host lineages at four sampling point (D0, D4, D15, and D27) during HTC. **(a)** Heatmap showing changes in the mean VHR of dominant mesophilic and thermophilic MAGs and their viruses shown in figure 4 panel **(b).** Data show mean ± SD with of three biological replicates per treatment (n=3), different lowercase letters between treatments denote for significant differences at $p < 0.05$.

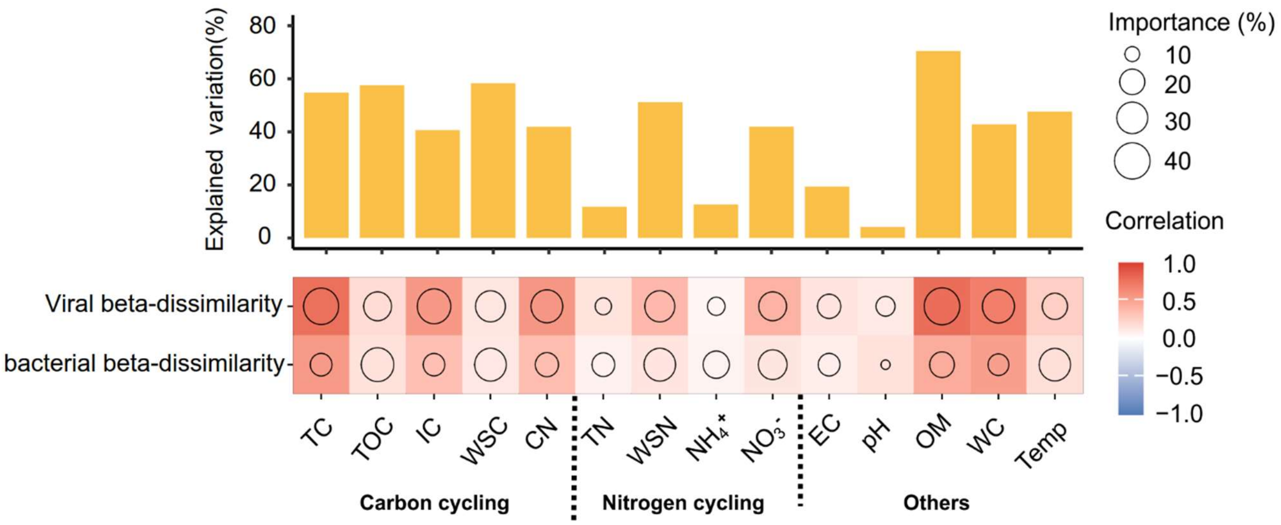

Fig. S15 Random Forest modelling comparing the potential importance of bacterial (b beta-dissimilarity) and viral community (v beta-dissimilarity) explaining the variation in nutrient turnover during HTC based on metagenomic datasets. Circle sizes and bar heights represent the proportion of explained variability of given composting properties calculated via multiple regression modeling and variance decomposition analysis. Circle colors represent the direction of Spearman correlations. The abbreviations of composting properties are following: total carbon content (TC); total organic carbon content (TOC); inorganic carbon content (IC); water soluble carbon (WSC); carbon-nitrogen ratio (CN); total carbon content (TN); ammonium ($NH_4^{+)}$ and nitrate (NO_3^-) concentrations; water-soluble nitrogen (WSN); temperature (Temp); total sulfur content (TS); water content (WC); organic matter content (OM).

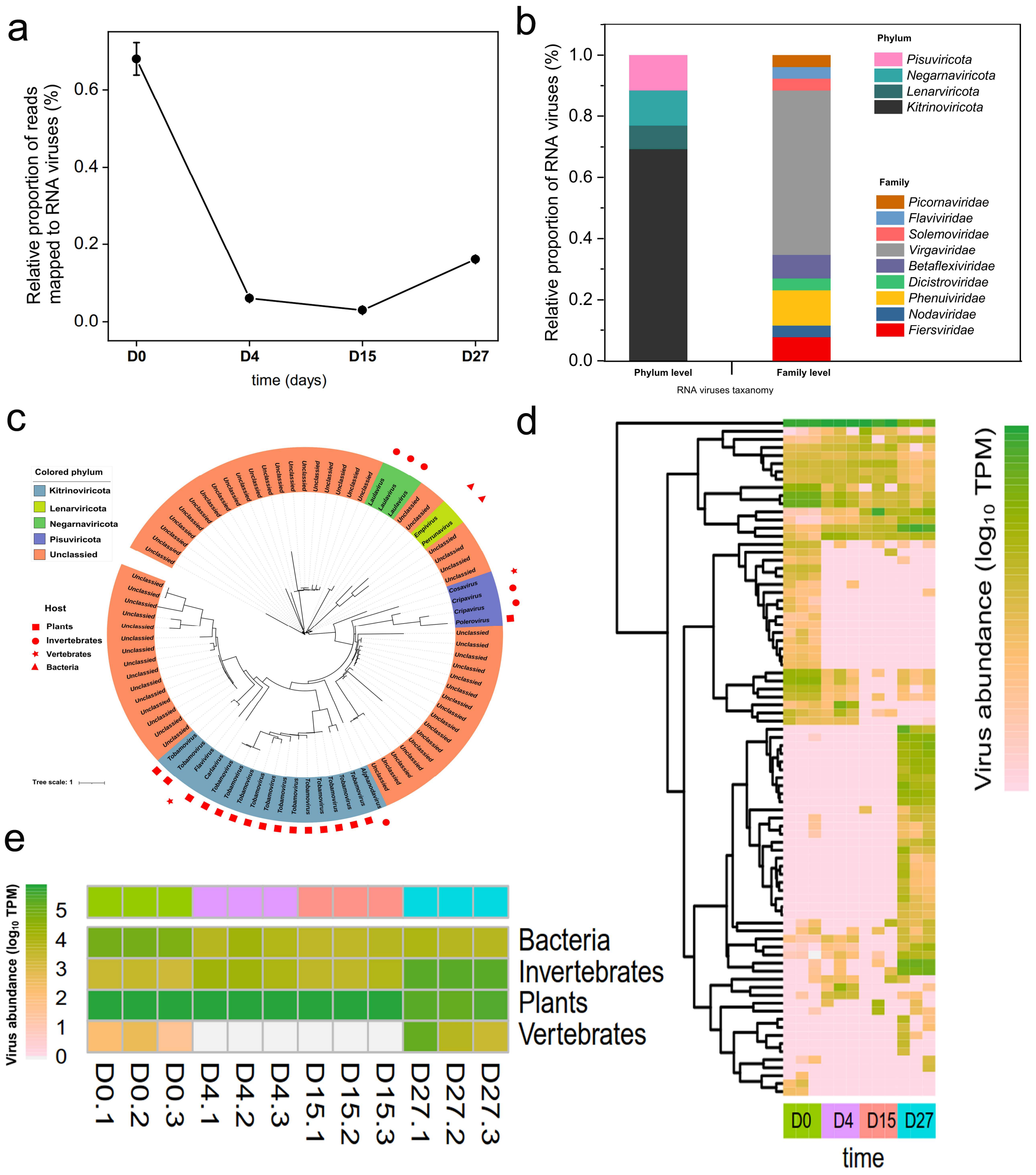

Fig. S16 Overview of RNA viruses obtained from the 12 composting metatranscriptomes. (a) the relative proportion of mRNA reads mapped to RNA viruses across different phase of composting. (b) Taxonomic composition of RNA viruses at phylum and family level across all phases of composting. (c) Phylogenetic relatedness and predicted host species group for four phyla of RNA viruses within the Riboviria realm, based on multiple sequence alignments of RdRP gene. (d) Temporal dynamics in relative abundances of all RNA viruses during the HTC composting; phylogeny on the Y-axis is based on all RNA viruses associated with bacteria, invertebrates, plants and vertebrates. (e) The relative RNA viral abundances during the HTC composting grouped by hosts types, including bacteria, invertebrates, plants, vertebrates.

Supplementary Data

Supplementary Data 1 | Detailed information of predicted viral genomes identified in the composting samples.

Supplementary Data 2 | Basic information, abundance, transcriptional activity and taxonomic affiliation of the dereplicated bacterial MAGs.

Supplementary Data 3 | Virus-host linkages predicted by shared tRNA, genomic matches with host genomes, and protospacer-spacer matches.

Supplementary Data 4 | Basic information, abundance, transcriptional activity and taxonomic affiliation of the dereplicated vOTUs.

Supplementary Data 5 | The presence and detailed functional description of genes associated with all MAGs.

Supplementary Data 6 | Detailed functional descriptions of auxiliary metabolic genes found in viral genomes identified by DRAM-v.

Supplementary Data 7 | Detailed accession numbers of metagenomic and metatranscriptomic reads of all composting samples.

Supplementary Data 8 | The non-redundant functional proteins in viral contigs were annotated using VIBRANT.
